# The composition of bacterial communities associated with plastic biofilms differs between different polymers and stages of biofilm succession

**DOI:** 10.1371/journal.pone.0217165

**Published:** 2019-06-05

**Authors:** Maria Pinto, Teresa M. Langer, Thorsten Hüffer, Thilo Hofmann, Gerhard J. Herndl

**Affiliations:** 1 Department of Limnology and Bio-Oceanography, Center of Functional Ecology, University of Vienna, Vienna, Austria; 2 Research Platform ‘Plastics in the Environment and Society’, University of Vienna, Vienna, Austria; 3 Department of Environmental Geosciences, Center for Microbiology and Environmental Systems Science, University of Vienna, Vienna, Austria; 4 NIOZ, Department of Marine Microbiology and Biogeochemistry, Royal Netherlands Institute for Sea Research, Utrecht University, Den Burg, The Netherlands; Loyola University Chicago, UNITED STATES

## Abstract

Once in the ocean, plastics are rapidly colonized by complex microbial communities. Factors affecting the development and composition of these communities are still poorly understood. Additionally, whether there are plastic-type specific communities developing on different plastics remains enigmatic. We determined the development and succession of bacterial communities on different plastics under ambient and dim light conditions in the coastal Northern Adriatic over the course of two months using scanning electron microscopy and 16S rRNA gene analyses. Plastics used were low- and high-density polyethylene (LDPE and HDPE, respectively), polypropylene (PP) and polyvinyl chloride with two typical additives (PVC DEHP and PVC DINP). The bacterial communities developing on the plastics clustered in two groups; one group was found on PVC and the other group on all the other plastics and on glass, which was used as an inert control. Specific bacterial taxa were found on specific surfaces in essentially all stages of biofilm development and in both ambient and dim light conditions. Differences in bacterial community composition between the different plastics and light exposures were stronger after an incubation period of one week than at the later stages of the incubation. Under both ambient and dim light conditions, one part of the bacterial community was common on all plastic types, especially in later stages of the biofilm development, with families such as *Flavobacteriaceae*, *Rhodobacteraceae*, *Planctomycetaceae* and *Phyllobacteriaceae* presenting relatively high relative abundances on all surfaces. Another part of the bacterial community was plastic-type specific. The plastic-type specific fraction was variable among the different plastic types and was more abundant after one week of incubation than at later stages of the succession.

## Introduction

Between 4.8 and 12.7 million metric tons of plastic are estimated to enter the ocean every year [1]. When plastics enter aquatic systems, a microbial biofilm rapidly develops on the plastic surface. The microbial community composition of this biofilm is typically different from that in the ambient water [2] and might play an important role in the fate of plastics in the ocean [3]. Biofilm development or biofouling can cause previously positively buoyant plastics to eventually sink into the water column [4, 5]. Also, the biofilm might include individual microbial species or consortia capable of degrading plastics [6]. Also, invasive and pathogenic species might be transported over large distances by plastic debris [7]. The composition of these plastic associated microbial communities is affected by spatial and temporal variations in environmental parameters [8, 9]. Comparative studies, however, following the development of the microbial biofilm on different plastic types in the same environment are scarce [9, 10].

Studies on the early biofilm formation (9-72h) on plastic surfaces have reported a community succession with typical biofilm primary colonizers of the *Proteobacteria* phylum, such as the genera *Alteromonas* (Gammaproteobacteria class) and *Roseobacter* (Alphaproteobacteria class) [11, 12], followed by members of the phyla *Bacteroidetes*, *Acidobacteria*, *Actinobacteria*, Cyanobacteria, *Firmicutes*, *Planctomycetes* and *Verrucomicrobia* [13].

Zettler et al. [14] used the term “plastisphere” to describe the specific microbial community developing on plastics collected in the surface waters of the North Atlantic and comprising microbes different from those found in the ambient water. Since then, several studies reported the existence of this plastic-specific microbial community in aquatic systems [15–21]. For instance, the fungal community composition of the biofilm developing on submerged wood differed from that on two plastics types (polyethylene-PE and polypropylene-PP) [16]. Also the bacterial community developing on PE sheets differed from that developing on a dolly rope at the seafloor [15]. Furthermore, a recent study using polystyrene (PS), PP and PE showed that the bacterial communities developing on the different plastic polymers were distinct from each other after two weeks and different from the communities developing on glass and cellulose [22]. This suggests that the type of polymers also influences the microbial community composition of the plastic associated biofilm.

It appears, however, that a specific plastic community does not always develop. Oberbeckmann et al. [9] found that a biofilm with a similar bacterial community composition was established on both PET bottles and glass slides incubated in three different seasons and locations. This suggests that typical biofouling processes were the main drivers for the biofilm formation rather than the type of surface. More recently, a study has shown that plastic-specific microbial communities only develop under particular environmental conditions [23]. Nevertheless, even in cases where no major differences in the microbial community composition between different surfaces were found, plastic-specific taxa were identified. Examples of plastic-specific taxa are members of the families *Alcanivocareacea* and *Cryomophaceaea* being specifically enriched on PET surfaces in comparison to glass [9], and members of the families *Hyphomonadaceae* and *Erythrobacteraceae* dominating on PE and PS surfaces over wood pellets [23]. The reasons for the occasional preferential attachment of certain taxa to plastic or specific plastic types remain enigmatic. Also, the role of environmental factors and physico-chemical properties of the plastics on biofilm formation is largely unknown.

Solar radiation has direct and indirect effects on marine microbial communities, usually suppressing bacterial activity and growth [24,25]. In surface waters, direct effects result from the deleterious effects of ultraviolet (UV) radiation on DNA, proteins and other macromolecules [26,27]. Indirect effects of solar radiation are UV-induced changes in the bioavailability of near-surface dissolved organic matter (DOM) due to photochemical transformations [28,29]. Bacterial communities in marine biofilms are impaired by solar radiation [25,29]. Partial plastic photo-oxidation typically precedes biotic plastic degradation by changing surface properties of the polymers such as roughness, topography and chemistry [3]. Hence, one might assume that the development of biofouling communities on marine plastics is also affected by solar radiation.

Solar radiation, particularly UV, generates free radicals during plastic photo-degradation processes and thereby changes the polymer properties [30] which, in turn, facilitates biodegradation processes (for a review on biological degradation of plastics see Shah et al. [31]). A recent study shows that plastics leach dissolved organic carbon into the surrounding seawater, regardless whether exposed to solar radiation or kept in the dark, stimulating heterotrophic microbial activity [32]. Furthermore, Royer et al. [33] reported the production of methane and ethylene if PE is exposed to natural solar radiation. Hence, solar radiation is likely affecting biofilm development directly via its effect on cellular constituents and indirectly by its effect on the plastics and organic matter.

In this study we addressed the question whether there is a general “plastisphere”, i.e., a plastic-associated microbial community independent of the plastic type, or whether there is a plastic type-specific community developing. Furthermore, we studied how biofilm successional stages and exposure to solar radiation might affect these plastic-associated microbial communities.

## Material and methods

### *In situ* incubations of different plastics at the sea surface

Virgin polyvinylchloride (PVC) pellets with approximately 5 mm diameter, containing two plasticizers, di(2-ethylhexyl) phthalate (DEHP) and di-isononyl phthalate (DINP), 4 cm^2^ sized pieces of virgin polypropylene (PP), LDPE and HDPE, with approximately 0.5 mm thickness, and glass slides of 1 cm^2^, with approximately 1 mm thickness, were mounted on a floating frame (S1 Fig) in the Northern Adriatic, approximately 500m off the coast of Rovinj, Croatia (S2 Fig) over a three-month period (November 2016—January 2017). LDPE, PP and PVC were purchased from Goodfellow Cambridge Ltd., while the HDPE consisted of pieces cut from an empty sodium chloride chemical container (Sigma-Aldrich Co. LLC). The glass pieces were cut from microscope slides (Carl Roth GmbH) and used as an inert control. Prior to incubating the plastics on the floating frame, all plastic and glass pieces were rinsed with 70% ethanol and Milli-Q water.

To assess the influence of solar radiation on the development and structure of the biofilm community, two different treatments were established. One set of plastic types and glass slides were exposed to the full range of solar radiation, while the other set of plastic surfaces and glass slides were held under dim light conditions. All the plastics and the glass slides were mounted on a custom-made rectangular floating frame of 1.5m x 0.5m (for a scheme see S1 Fig). The frame supported four metal wires where the plastic pieces and the glass slides were attached by a thin nylon string thread through a small hole on the tip of each piece of plastics or glass. To study microbes from approximately the same surface area of each material, we mounted four 1 cm^2^ glass slides on each string. These four pieces were regarded and treated as one single sample. For all the PVC samples, 10 pellets were considered as one sample. For the other plastics, one piece was considered as one sample. The different plastics and glass slides were equally distributed between the metal wires, with a spacing of around 8 cm between them. To achieve dim light conditions, two of the four wires supported two grey tubes (10 cm inner diameter) that shielded off the plastics and glass slides from direct solar radiation. The metal wire ran through the center of each tube and care was taken to ensure that the different surfaces did not touch the tube walls or each other. The tubes were open on both sides to allow water exchange and maintaining dim light conditions. The frame was connected to a single main float and therefore, oriented itself to the direction of the currents, allowing constant water flow through the tubes.

Three samples of each plastic type and glass from each treatment were collected after one week (November 2016), one month (December 2016) and two months (January 2017) of incubation. To remove organisms not part of the biofilm, samples were carefully rinsed 4 times with 0.2 μm freshly filtered seawater. Two of the three samples collected from the same wire were immediately frozen at -80°C for DNA extraction. The third sample was immersed in 2% glutaraldehyde for 10 min and then stored at -80°C for scanning electron microscopy (SEM). For comparison, surface water samples were also taken in close vicinity to the floating frame at each sampling day. Seawater was filtered in duplicates (two times two L) onto 0.2 μm pore-size Millipore (Merck KGaA, 47 mm filter diameter) GTTP filters and stored at -80°C for DNA extraction. Additionally, plastics from the dark treatment were also collected to measure DEHP and DINP concentrations in the plastics. Additive measurements were done as described in the Supporting Information (S1 File).

### DNA extraction and amplification

DNA from plastic and glass pieces and filters (for ambient water) was extracted using a modified bead-beating approach in combination with the Puregene Tissue DNA extraction kit (Qiagen, Valencia, CA) (as described in S2 File of the Supporting Information) [34]. To compare the bacterial community of the biofilm with that of the ambient water, PCR amplification of the 16S rRNA V3-V4 hypervariable region was used with the primer pair 341F (5´- CCT ACG GGN GGC WGC AG-3´) and 802R (5´- TAC HVG GGT ATC TAA TCC-3´) resulting in fragments of ~460 bp. Each PCR was performed in a total volume of 52 μL containing 25 μL Library Amplification Ready Mix 2xMM (Kapa Biosystems), 1 μL of each 25 μM primer, 23 μL of sterile water and 2 μL of extracted DNA. Amplification was done on a Mastercycler Pro (Eppendorf AG, Germany). The conditions consisted of an initial denaturation at 95°C for 3 min, followed by 20 cycles at 98°C for 20 s, at 56°C for 30 s and at 72°C for 30 s, and a final elongation at 72°C for 5 min.

PCR products were then purified in a 96-well plate using the Agencourt AMPure XP Purification kit and protocol (Beckman Coulter Life Sciences). These purified PCR products were barcoded and an additional 10-cycle PCR was done, followed by paired-end next-generation amplicon sequencing (2 x 250 bp) using Illumina MiSeq technology at Microsynth AG, Switzerland. All raw sequence files have been submitted to the NCBI Sequence Read Archive (SRA) database (BioProject: PRJNA515271).

### Processing of sequence data

Sequence data were processed with Usearch tools by merging paired reads, quality filtering (max. error 1 in 100 nucleotides), eliminating singletons, chimera filtering, mapping, and operational taxonomic unit (OTU) clustering. OTU clustering was performed using the UPARSE OTU clustering Algorithm with USEARCH command lines [35]. For taxonomy assignment blastn and the SILVA_123_SSURef reference database were used (97% similarity between query and target sequences as threshold). Furthermore, the merging of taxonomy and OTU reads was done in Python. After quality filtering and phylogenetic classification, samples with less than 1000 reads were excluded from further analysis (one PVC DINP sample of the dim light treatment collected after one month and one after two months of incubation, one LDPE sample of the dim light treatment and one seawater sample both collected after two months of incubation).

### Phylogenetic diversity analysis

Shannon and Simpson diversity and OTU richness indexes were calculated through the iNext package in R [36] using coverage based rarefaction to a coverage of 97%. Analysis of variance (ANOVA) for each index was performed to identify differences between surface type, time points and treatments (n = 74, degree of replication = 2). To decipher differences in bacterial community composition between the different plastic types and seawater, post-hoc Tukey tests for each index were performed.

### Analysis of the bacterial community composition

OTU read numbers per sample were converted into relative abundances. Permutational multivariate analysis of variance (PERMANOVA) was used to compare the community composition between different surface types, time points and treatments (n = 74, degree of replication = 2). To understand whether the exposure treatment or the type of polymer influenced the presence and relative abundance of specific bacterial taxa, we performed PERMANOVA analysis using the Jaccard index and Bray-Curtis dissimilarity as distance measurements, respectively. The seawater samples were excluded from these analyses.

Non-Metric Multidimensional Scaling (NMDS) with Bray-Curtis Dissimilarity as a distance measurement was conducted for a visual representation of the similarity of the bacterial community composition between samples. To determine differences in the community composition of heterotrophic bacteria between the treatments and plastic types, the PERMANOVA and NMDS analyses were repeated after excluding *Cyanobacteria*.

### Determining surface discrimination of specific bacterial taxa

To identify bacterial taxa discriminating specific surfaces, we performed a linear discriminant analysis size effect (LEfSe) for each month. The analysis was performed on the online Galaxy framework. Treatment was used as a subclass in the analysis to assure that differences between the surface types were consistent within each treatment. The default settings were used for all other parameters, for data formatting and LDA effect size analysis. Individual LEfSe analyses were also performed to identify bacterial families discriminating for each of the plastics compared to glass. Treatment was also used as a subclass in these analyses, but due to the large difference in relative abundance of the different families between the ambient and dim light treatments growing on the same surface, the alpha value for the factorial Kruskal-Wallis test among classes was set to 0.5. The default settings were used for all other parameters.

To determine the most abundant bacterial families discriminating a specific plastic type in comparison to glass, we applied the following formula for all families with a relative abundance of > 5% of the total community in at least one of the samples:

$$i_{F}=\left(\frac{P_{F}-G_{F}}{P_{F}+G_{F}}\right)*100$$

Where *P_F_* is the relative abundance of family *F* on plastic type *P* and *G_F_* is the relative abundance of family *F* on glass. *i_F_*, the discrimination factor of family F, was calculated separately for each treatment and each month. All non-identified cyanobacterial families were grouped in one single Cyanobacteria group.

### Statistical analysis

All statistical tests and plots were conducted using R Studio Version 1.0.153 (RStudio, Inc. 2009–2017) unless stated otherwise.

### SEM analysis

SEM samples were dehydrated in an ethanol dilution series of 30%, 50%, 70%, 80%, 90%, 95% each for 10 min and three times in 100% absolute ethanol for 10 min. The dehydrated samples were CO_2_ critical point dried with a CPD 300 auto critical point dryer (Leica Microsystems). The dried pieces were gold-coated using a JFC-2300HR sputter coater (JEOL Ltd.) for 80s. Pictures were taken using a secondary electron detector with a JEOL JSM-IT300 scanning electron microscope at 150x, 450x, 2000x magnification, 15kV acceleration voltage in ultra-high vacuum.

## Results

After one week of incubation, the surface of PVCs exhibited a more developed biofilm in both the ambient and dim light treatment compared to the other plastics and the glass slides (Figures A-F from S3 File), with a larger area of their surface covered by a biofilm matrix and individual cells. After two months of incubation, all plastics and glass surfaces were patchily covered with a biofilm (Figures A-F from S3 File). Diatoms were found on all plastics of the ambient light treatment throughout the incubation period. Although diatoms were also found in the biofilm on all the plastic and glass surfaces even under dim light conditions, they appeared to be considerably less abundant in the latter (Figures A-F from S3 File). The concentrations of DINP in the PVC DINP pellets ranged between 33% and 37% (w/w) over the incubation period, while the concentration of DEHP in the PVC DEHP pellets was around 41% throughout the incubation. LDPE, HDPE and PP did not contain measurable phthalate concentrations.

**Fig 1:**
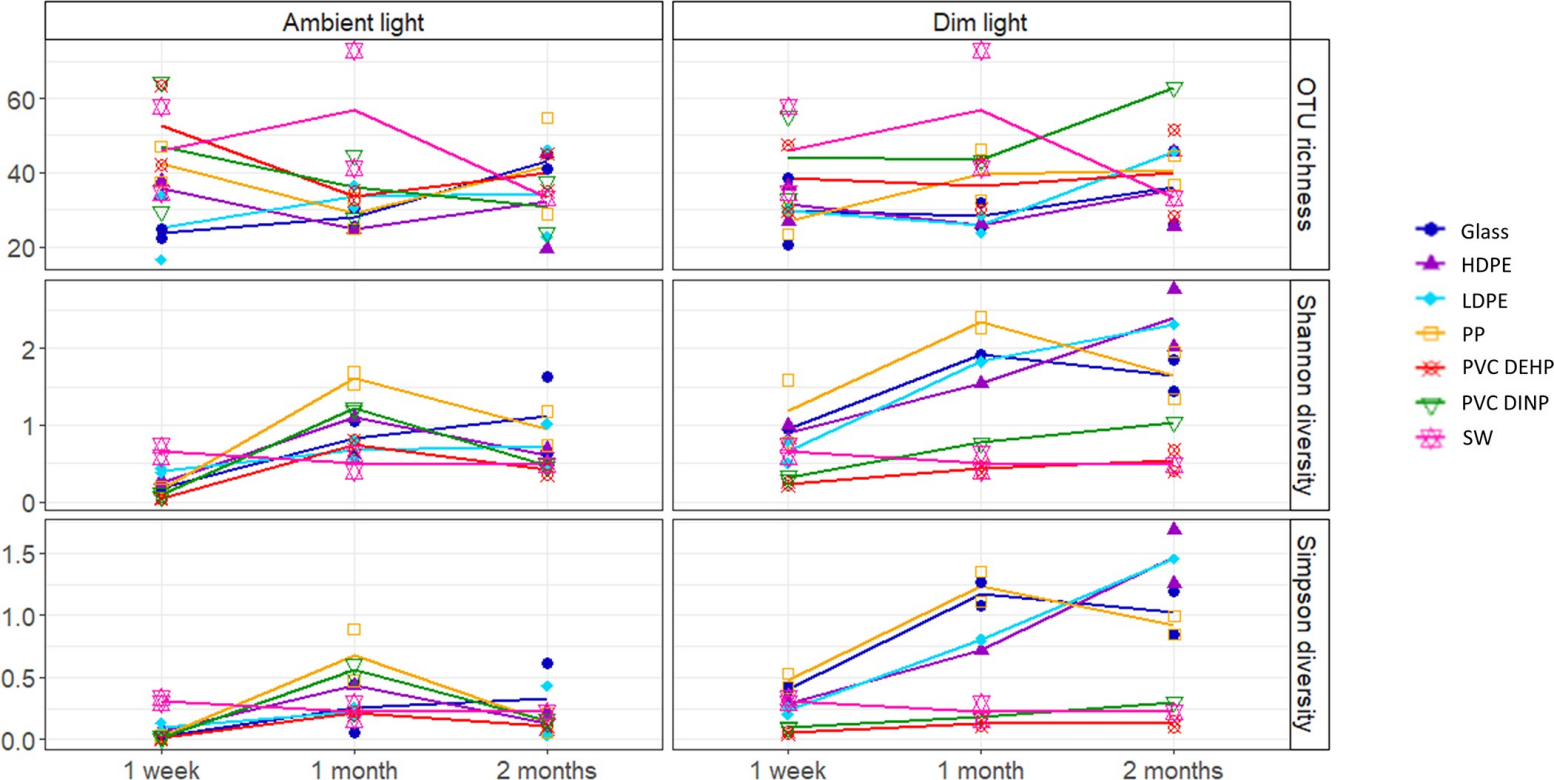
OTU richness and diversity of the bacterial community of the biofilm of all plastics, glass and water samples after one week, one month and two months of incubation. Each symbol represents a sample. Duplicate samples from the individual surfaces and seawater were collected except LDPE after one month incubation under dim light conditions, seawater after two months incubation and PVC DINP under dim light conditions after one and two months of incubation. The lines connect the middle value between the duplicates of the different sampling points. In cases where there were no duplicates, the lines connect to the value of the single sample.

A total of 4823 OTUs was classified and used for comparative analyses, from which 3303 OTUs were present after one week of incubation, 4277 OTUs after one month and 3921 OTUs after two months of the incubation. OTU richness and diversity varied among light treatments, sampling time and surface type (S1 Table). In the ambient light treatment, OTU diversity and richness of the biofilm communities were similar among all plastic surfaces and glass. In the dim light treatment, both PVCs exhibited a high OTU richness but low diversity in all sampling months. PP and glass exhibited the highest bacterial diversity after one week and one month of incubation, indicated by both the Shannon and Simpson index, but after two months of incubation LDPE and HDPE exhibited a higher bacterial diversity than the other surfaces (Fig 1, S4–S6 Files).

OTU richness and diversity were significantly higher in the dim than the ambient light treatment (S1 Table) over the two months of incubation (PERMANOVA: F = 3.176, p = 0.001) (Fig1).

### Bacterial community composition

**Fig 2:**
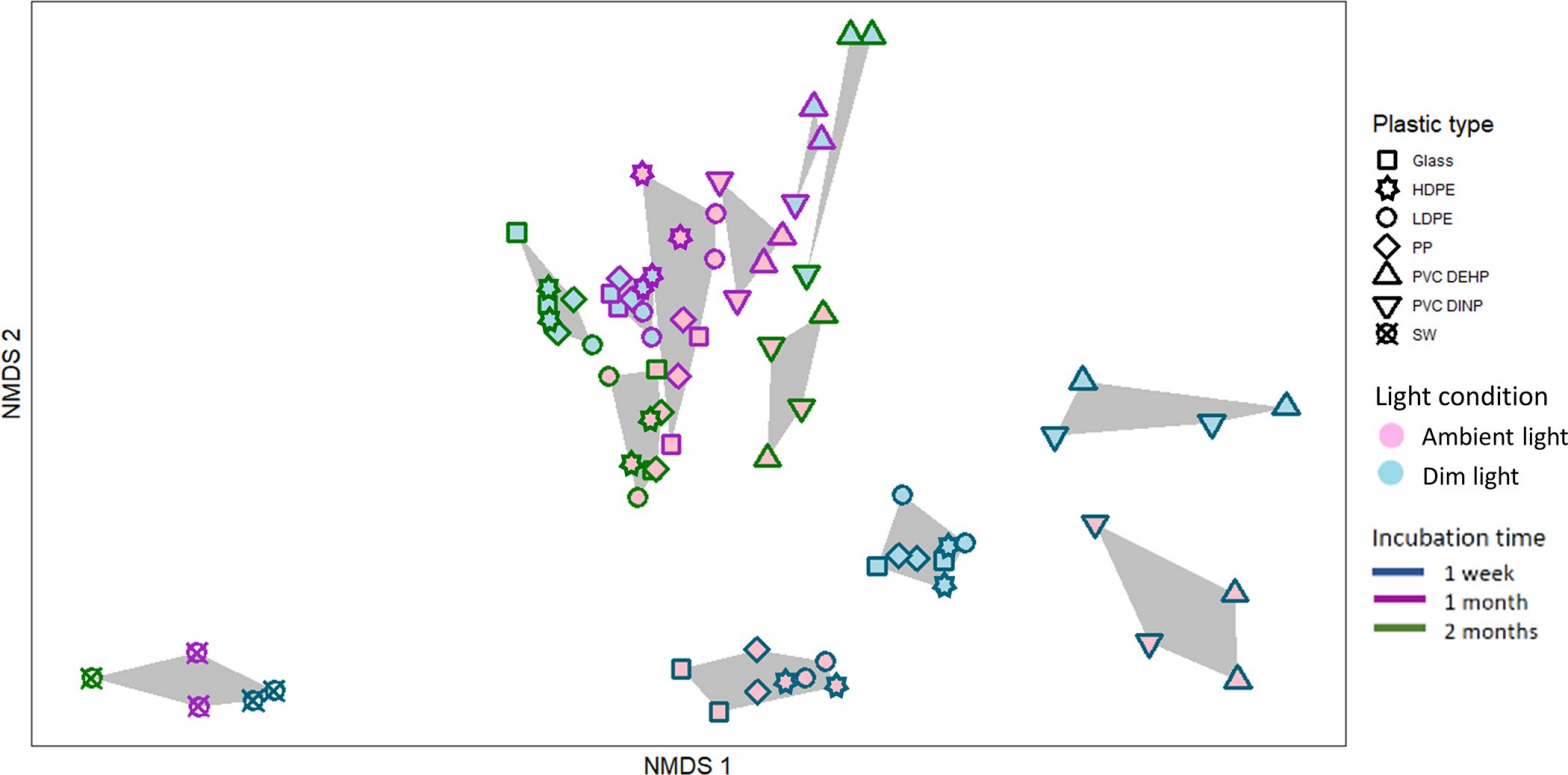
NMDS representation of the similarity between the bacterial community composition of the biofilm on different surfaces and seawater. Bray-Curtis Dissimilarity was used as the distance measurement. Each point represents one sample. The grey polygons connect groups of samples from each time point exposed to the same light conditions.

**Fig 3:**
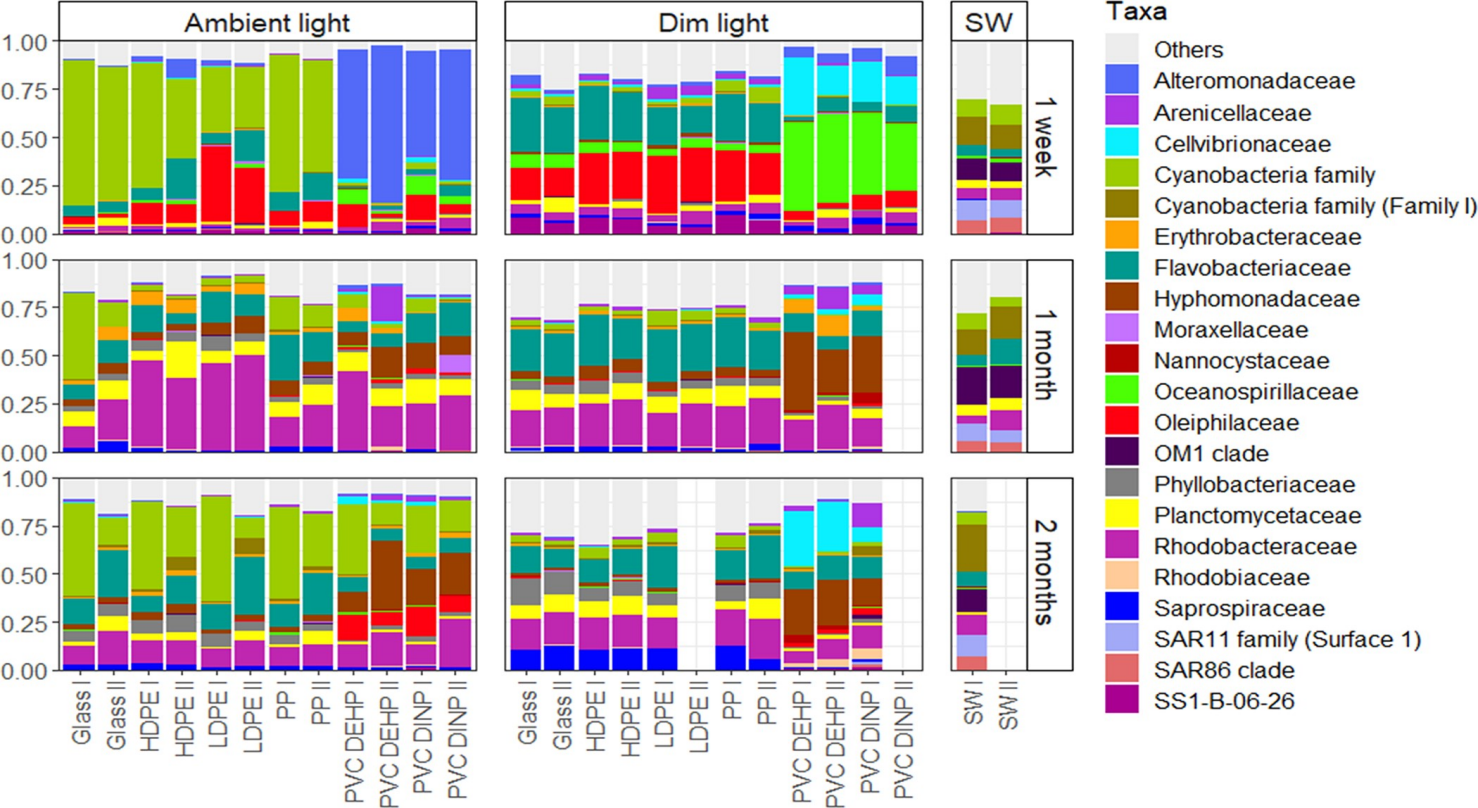
Relative abundances of the bacterial families representing at least 5% of the total bacterial community in at least one sample. Families representing < 5% of the total bacterial community were grouped into the category “Others”.

The composition of the bacterial community differed between the type of surface (i.e., different plastic types and glass) (PERMANOVA: F = 16.11, p = 0.001), light regime (PERMANOVA: F = 39.33, p = 0.001) and duration of incubation (PERMANOVA: F = 48.07, p = 0.001) when considering both, the entire bacterial community and solely the heterotrophic bacterial community (S2 Table). The influence of exposure and polymer type on the bacterial community composition was significant (p-values < 0.05) after one week, one month and two months of incubation, both in terms of presence/absence of organisms (S3 Table) and their relative abundances (S4 Table). Even though we only had duplicate data per sampling, the NMDS analysis (Fig 2) and the relative abundances of the most abundant families (Fig 3) indicate a clustering of the samples in three groups with different bacterial community composition. The community developing on the PVCs formed a distinct group and the communities developing on the other surfaces (glass, LDPE, HDPE and PP) formed another cluster. This clustering was stronger after one week of incubation and, overall in the dim light treatment. After one month of incubation all samples from the ambient light treatment exhibited relatively similar communities (Figs 2 and 3). Moreover, all the bacterial communities associated with plastic and glass surfaces were different from that of the ambient water (Fig 2, S3 Fig).

After one and two months of incubation, the communities developing on the different surfaces were more different from each other when surfaces were exposed to ambient solar radiation than under dim light conditions (Fig 3). This difference appeared to be diminished, however, when considering only the heterotrophic bacterial community (S3 Fig).

### Community composition after one week

Differences between the bacterial communities associated with the PVC and the other plastic and glass surfaces were most pronounced after one week of incubation under both dim and ambient light conditions, and decreased with increasing duration of the incubation (Fig 3). After one week of incubation, PVC surfaces were highly dominated by families of Gammaproteobacteria, mainly *Alteromonadaceae*, *Cellvibrionaceae* and *Oceanospirillaceae*. The other plastic surfaces were mainly dominated by members of the phylum Cyanobacteria and of the families *Flavobacteriaceae* and *Oleiphilaceae* (Fig 3).

Under ambient light conditions, the community associated with the PVCs was dominated by one OTU affiliated to the genus *Alteromonas* with relative abundances ranging from 54% to 81% of the total 16S rRNA gene abundance (S4 Fig). Under dim light conditions, this OTU was present in much lower relative abundance on PVC reaching at most 7% in one of the PVC DINPs. On all other surfaces, this OTU was present only in very low relative abundances. These non-PVC surfaces were dominated by Cyanobacteria reaching relative abundances of 75% of the total 16S rRNA gene abundance on glass slides, while an uncultured bacterium of the *Oleiphilus* genus showed a clear discrimination for LDPE with relative abundances of up to 39% of the total bacterial community (S4 Fig).

**Fig 4:**
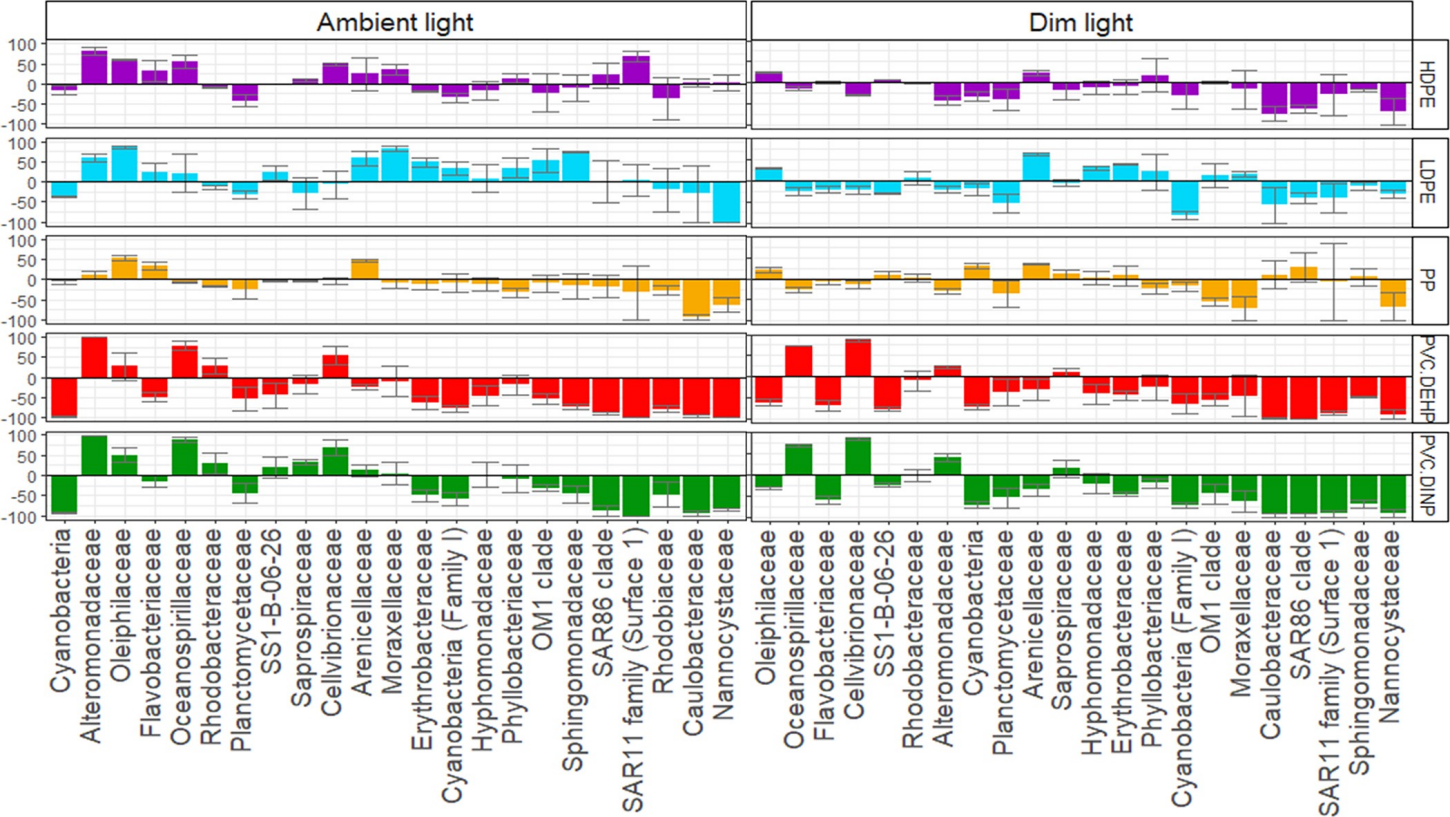
Values of *i_F_* representing the discrimination of the most abundant bacterial families (with >5% relative abundance in at least one of the samples) for either one of the plastics or glass after one week under ambient and dim light conditions. Positive values indicate discrimination for a specific plastic type over glass, negative values indicate discrimination for glass over a specific plastic type. Bacterial families are ordered from high (left) to low (right) relative abundance. Total abundance was determined per treatment and time point. The LEfSe analysis identifying discriminating families of either glass or of any of the polymers after one week of incubation can be found in S5 Table.

The bacterial community of the PVCs in the dim light treatment was dominated by four OTUs, two were affiliated with the family *Cellvibrionaceae* and two with the family *Oceanospirillaceae*. One of the *Cellvibrionaceae* OTUs, classified as the genus *Aestuariicela* (OTU 15), was relatively more abundant on the PVC DINP than on other surfaces (S4 Fig), with a relative abundance of approximately 1% of the total 16S rRNA gene. Nevertheless, all three families, *Oceanospirillaceae*, *Cellvibrionaceae* and *Alteromonadaceae*, exhibited a relatively higher abundance on PVCs than on glass in both light treatments (Fig 4, S5 Table). In contrast, most of the other families, including *Planctomycetaceae*, *Flavobacteriaceae*, *Cryomophaceae* among many others, dicriminated for glass over either one of the PVCs (S5 Table). While families discriminating for both PVCs over glass were the same in both dim and ambient light treatments, families that discriminated for any of the other plastics (PEs and PP) over glass were different between the dim and ambient light communities. *Oleiphilaceae* and *Arenicellaceae* families discriminated for LDPE, HDPE and PP over glass in both light treatments, while other families discriminated for one of the surfaces in only one of the treatments. For example, the *Alcanivoracaceae* family was relatively more abundant on the LDPE than on glass when exposed to dim light while when exposed to ambient light, discrimination for LDPE was not apparent (Fig 4).

**Fig 5:**
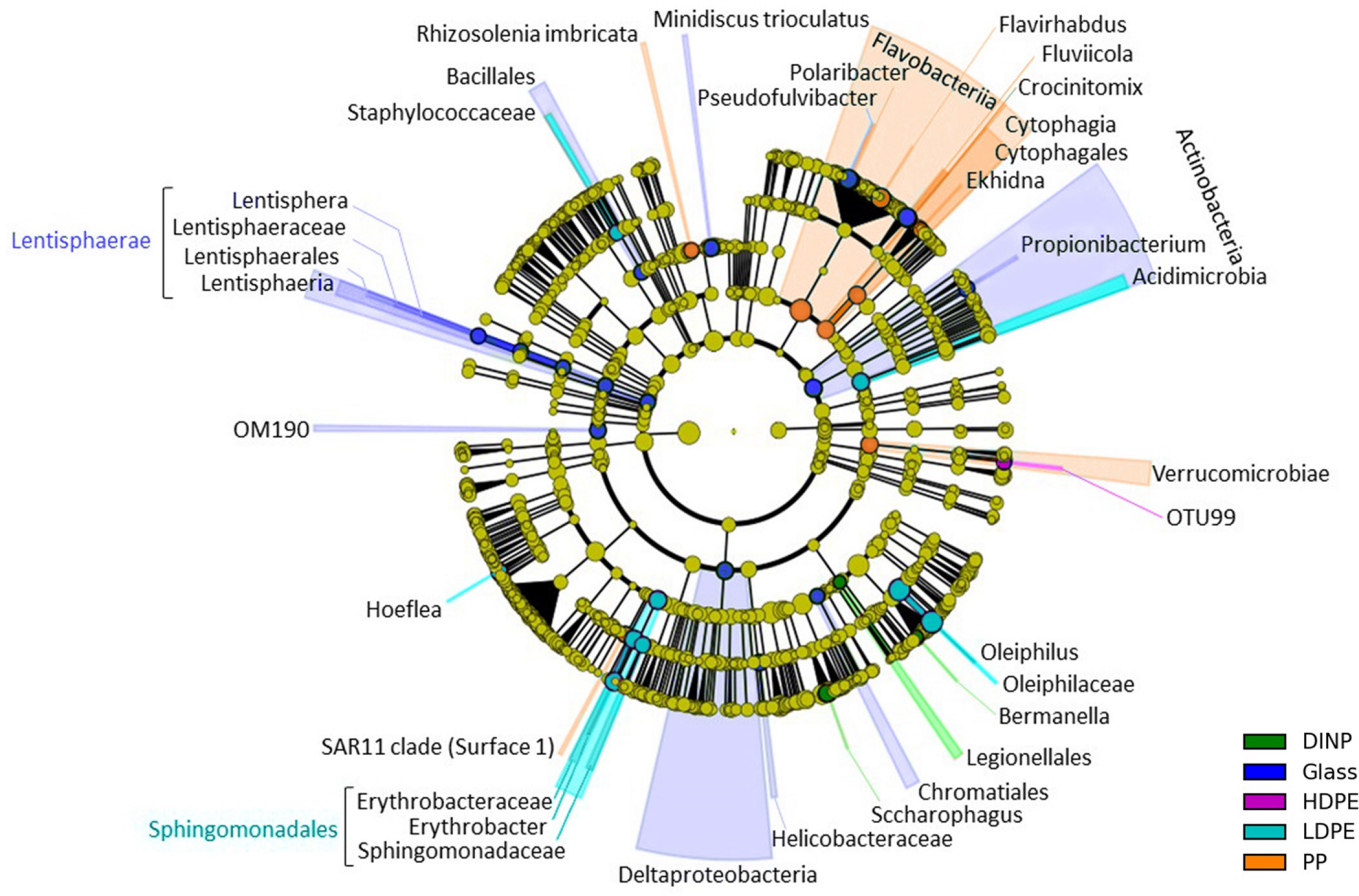
Taxa that discriminated for one of the surfaces over all other surfaces considering both light treatments and determined by the discriminant linear analysis (LEfSe) after one week of incubation. The results of the LEfSe analysis can be found in S8 Table.

After one week, the phyla *Lentisphaerae* and *Actinobacteria* and the class *Deltaproteobacteria* were favoring glass against all other surfaces. The phyla *Flavobacteria* and *Verrucomicrobiae* significantly discriminated for PP and the order *Sphingomonadales* and the family *Oleiphilaceae* for LDPE (Fig 5).

### Community composition after one month

After one month of incubation, differences between the community compositions among the different surfaces decreased, with the families *Hyphomonadaceae*, *Flavobacteriaceae*, *Rhodobacteraceae*, *Planctomycetaceae* dominating all surfaces. Additionally, Cyanobacteria were relatively abundant on glass and PP in the ambient light treatment. Both LDPE and HDPE exhibited relatively high abundances of OTUs affiliated with the genus *Loktanella* (OTU 9) and *Ruegeria* (OTU 12) (S4 Fig). The relatively abundant *Rhodobacteraceae* family discriminated for HDPE and *Sphingomonadaceae* for LDPE over glass in both light treatments, while all the other families showed no discrimination for either HDPE, LDPE or PP in comparison to glass (Figure A in S7 File, S6 Table). Contrary to the previous month, *Oleiphilaceae* discriminated for PVC DINP instead of LDPE (Figure A in S8 File). Some fairly abundant families, however, did show discrimination for glass in only one of the light treatments over one of the three plastics. For instance, the families *Saprospiraceae* and *Rubritaleaceae* discriminated for glass over LDPE in the ambient light treatment (Figure A in S8 File). Some families, however, still showed a strong discrimination for the PVCs over glass and *vice versa*. From the most abundant families, *Hyphomonadaceae*, *Oleiphilaceae*, and *Cellvibrionaceae* discriminated for both types of PVC over glass and additionally, *Alteromonadaceae* and *Sphingomonadaceae* also discriminated for PVC DEHP over glass. *Phyllobacteriaceae*, *Saprospiraceae*, Cyanobacteria, the OM1 clade and *Thiotrichaceae* discriminated for glass over both PVCs (Figure A in S7 File, S6 Table). Additionally, the OCS116 clade also discriminated for glass over PVC DEHP. The relatively abundant family *Rhodobacteraceae* discriminated for HDPE over glass (S6 Table), especially in the ambient light treatment (Figure A in S7 File).

### Community composition after two months

After two months of incubation, most of the taxa showing discrimination for a specific plastic type were relatively low in abundance, except for the abundant *Flavobacteria* discriminating for LDPE (Figure B in S8 File).

Under ambient and dim light conditions both PVCs exhibited a higher relative abundance of the family *Hyphomonadaceae* and a lower relative abundance of the family *Phylobacteriaceae* than the other surfaces. On both PVCs in the dim light treatment, the family *Cellvibrionaceae* were more abundant than on the other surfaces (Fig 3).

After two months of incubation, the families *Hyphomonadaceae* and *Oleiphilaceae* still discriminated for both PVCs over glass in both light treatments and, similar to after one month of incubation, *Cellvibrionaceae* and *Oceanospirillaceae* maintained the discrimination for PVC DEHP over glass (Fig 3). The family *Nannocystaceae* discriminated for glass over HDPE and PP, and the family *Arenicillaceae* discriminated for glass over HDPE (Figure B in S7 File, S7 Table).

## Discussion

### Role of plastic type in determining bacterial community composition

Apparently, the surface type has a stronger influence on the initial bacterial colonization and consequently, on the bacterial community composition, than on late successional stages (Figs 2 and 3). This is indicated by the more pronounced differences in bacterial community composition between polymer surfaces at the beginning of the incubation experiment than after one and two months of incubation (Figs 2 and 3). In the later successional stages of biofilm development, such as after one month and two months in our experiment, the bacterial community was likely more influenced by internal biofilm processes than by surface-related characteristics. The initial colonizers are in direct contact with the surface and hence are more likely to be influenced by its physico-chemical properties than bacteria embedded in an already developed biofilm.

Physico-chemical properties of surfaces, such as roughness, hydrophobicity, topography, surface free energy, charge and electrostatic interactions are known to influence the attachment of initial colonizers and biofilm development [3,37–39]. Hydrophobicity has been shown to determine initial bacterial attachment, with bacteria preferentially attaching to more hydrophilic surfaces [37–39]. Glass is highly hydrophilic compared to plastics, however, bacterial OTU richness and diversity of the communities developing on glass appear similar to those of plastics throughout our incubations (Fig 1). Actually, in the dim light treatment after one week of incubation, the glass biofilm appears to exhibit a higher bacterial diversity than both PVCs (Fig 1). Even though the bacterial biofilm was more developed on the PVCs after one week of incubation than on the other surfaces, this was not the case for the other plastic surfaces compared to glass. As we did not measure surface hydrophobicity, we cannot exclude that it might have influenced the biofilm bacterial community, however, our results suggest that there are other important factors affecting initial biofilm development on plastics.

When in contact with seawater, surfaces sorb organic and inorganic substances creating a conditioning layer that might attract or repel microorganisms [3]. The chemical composition of this layer is influenced by the properties of the solid surface. Rochman et al. [40] found that HDPE, LDPE and PP sorbed higher concentrations of organic pollutants than PVC and PET over a 12-month period. It also appears that the chemical composition of this conditioning layer is more relevant for initial bacterial attachment than other surface-related properties such as roughness and hydrophobicity [41]. The large differences in bacterial community composition after one week of incubation between the PVCs and the other surfaces might be an indication that differences in the chemical composition of the conditioning layer of the different surfaces play a selective role in the attachment of bacterial populations.

While OTU richness of the bacterial community on PVCs was higher than that of the other surface communities in both light treatments (Fig 1), OTU diversity was lower. This high richness and low diversity can be explained by the presence of very dominant OTUs, such as the genus *Alteromonas* in the biofilm of both PVCs under ambient light conditions, and OTUs belonging to the *Cellvibrionaceae* and *Oceanospirillaceae* families in the biofilm under dim light conditions.

Phthalates, such as DINP and DEHP are known to impact bacterial growth and development [42]. Iwaki et al. [43] found that Bacteria closely related to the families *Alteromonadaceae* and *Oceanospirillaceae* were capable of growing on phthalates, while other bacterial taxa have been shown to be inhibited by certain phthalates [42]. These two families discriminated for the two PVCs over glass after one week of incubation and the latter family discriminated for PVC DEHP over glass throughout the entire duration of the experiment (S8–S10 Tables). Thus, phthalates might act as a selection factor in the development of bacterial biofilms on PVCs [44]. This could also explain why the bacterial community composition in the PVC-associated biofilm remained different from that on glass even after one and two months of incubation (Fig 2), with some bacterial taxa discriminating for either one of the surfaces (Fig 4, S8–S10 Tables). For example, relatively abundant taxa such as Cyanobacteria, and families *Phyllobacteriaceae*, *Planctomycetaceae* and *Saprospiraceae* showed a consistent discrimination for glass over PVC in almost all samples (Fig 4, S5–S7 Tables). It has been suggested that surface properties are a selective factor for microbial biofilm initiation, hence surface degrading taxa are only selected during the initial successional stages of bacterial aggregate formation [45]. As the biofilm develops, the community is more structured via the dynamic interactions within the biofilm community, rather than by the selective pressure caused by the properties of the colonized surface [45]. This might explain the higher relative abundance of potential phthalate degraders after one week than after one and two months of incubation (Fig 3).

It is important to note, however, that our knowledge on the effect of DEHP and DINP on the above-mentioned bacterial taxa and on marine microbial communities is generally rather limited. Furthermore, whether members of the *Alteromonadaceae* and *Oceanospirillaceae* families as prominent members of these biofilms are indeed taking up phthalates is unknown and other plastic type related properties as discussed above might be responsible for their high abundance. We did not investigate the presence of other additives in the plastics we used. Different polymer types have typically different additives. Thus, differences in the composition and concentration of additives might have also be partly responsible for the compositional differences in the bacterial community composition on the different plastic types.

Oberbeckmann et al. [9] found that *Flavobacteria* and *Crocinitomix* discriminated for PET over glass after 5–6 weeks of incubation in the North Sea. In our study, *Flavobacteria* and *Crocinitomix* were discriminating for PP over all other surfaces after one week of incubation (Fig 5). *Flavobacterium* sp. has been associated with PP degradation [46], and *Cryomorphaceae*, to which *Crocinitomix* is affiliated, are known to degrade hydrocarbons [47]. *Oleiphilus* clearly discriminated for LDPE and is capable of degrading hydrocarbons [48] thriving in the presence of oil [49]. *Erythrobacter*, also discriminating for LDPE over all other surfaces, is also known to thrive in the presence of hydrocarbons [50]. This indicates that the polymer type and additives might play a regulatory role in the initial attachment and development of specific bacterial taxa on certain plastics.

Some studies report the existence of a plastic-specific community different from that in the surrounding seawater [8,14,51,52] and on detrital particles [22,51]. In contrast, other studies have not found any significant differences between communities developing on different plastics and other organic and inorganic surfaces [9,52]. In our study, the lack of major differences in the bacterial community composition between glass, HDPE, LDPE and PP is in agreement with studies comparing the development of the biofilm on plastics and other surfaces [9,10] that suggest that general biofilm processes rather than polymer-type associated characteristics are the main drivers of biofilm establishment and development in late biofilm development stages. Our study, however, also suggests that there is a small fraction of the bacterial community which is polymer type-specific particularly in the initial phase of the biofilm succession. These findings are in agreement with a recent study [18] where only a small percentage of the prokaryotic community developing on plastic polymers was different from that on glass, while a common core community dominated on all surfaces.

Even though we only studied duplicates, not allowing a more thorough statistical analysis, a consistent difference between the PVCs and the other surfaces is obvious throughout the entire incubation period, especially in the dim light treatment (Figs 2 and 3). Thus, polymer characteristics apparently influence the bacterial community composition to a certain extent.

### Influence of solar radiation on the microbial community composition

As expected, one of the major differences in bacterial community composition between the two light treatments was the high abundance of autotrophs, mainly Cyanobacteria, in the ambient light treatment in contrast to their very low abundance in the dim light treatment (Fig 3) [53]. Moreover, SEM analysis revealed that eukaryotic autotrophs such as diatoms and algae were also less abundant in the dim than the ambient light treatment. Heterotrophic bacterial metabolism is tightly linked to the composition and availability of organic matter [54]. Wagner et al. [55] suggested a shift in organic matter utilization by the bacterial community of a stream biofilm with changing light intensity, shifting from the utilization of photoautotrophically derived organic matter at high light intensities to more complex organic matter under low light availability. Furthermore, it appears that under low light conditions biofilm communities utilize mostly allochthonous organic carbon while shifting to autochthonous carbon at high light intensity [56].

The interaction between the direct effect of light on the availability of autochthonous organic carbon and the differences in relative abundance of photoautotrophs between the two light treatments might have contributed to the differences in bacterial community composition between the two light treatments.

Members of the *Alteromonadaceae*, such as *Marinobacter* and *Alteromonas*, known to thrive under solar radiation in the presence of oil [57,58], were highly abundant on the PVCs after one week in the ambient light treatment (Fig 3). Their relative resistance to UV radiation together with their potential ability to degrade hydrocarbons could have given them a selective advantage over other bacteria in the ambient light treatment. However, whether these organisms do indeed degrade PVC in the environment remains to be shown. Furthermore, the indirect effect of solar radiation on the plastic associated biofilm and the influence of potentially different eukaryotic communities in the two light treatments, both not addressed in this study, might have also contributed to the observed differences in the biofilms developing under dim and ambient light conditions.

Solar radiation can have different effects on different types of plastics [30]. For instance, emission rates of hydrocarbon gases are higher from LDPE than from HDPE, PP, polyethylene terephthalate (PET), polycarbonate (PC), acrylic (AC) and polystyrene (PS), when exposed to solar radiation [33]. This can indirectly affect the community composition attached to different plastics. Romero-Castillo et al. [32] report higher release rates of dissolved organic carbon from HDPE, LDPE and PP when exposed to solar radiation as compared to plastics held in the dark. Furthermore, in the same study, the plastic leachates influenced bacterial abundance and activity differently, depending on the type of plastics and light conditions. Also, Paluselli et al. [59] noted differences in the leaching of additives from different plastics kept under different light conditions and in the presence or absence of microorganisms. This supports the idea that solar radiation has a significant impact on microbial communities by indirectly affecting compounds released from different polymers.

Solar, and particularly ultraviolet (UV), radiation is also known to exert varying effects on different microorganisms [60]. The decreasing differences in the composition of the bacterial communities developing under ambient and dim light conditions after one month of incubation compared to after one week of incubation might be the result of the increase in biofilm thickness, shielding off harmful UV radiation from a substantial part of the community.

The impact of light exposure on biofilm communities in the marine environment is highly understudied, and to our knowledge this is the first study shedding light onto the impact of solar radiation on the biofilm developing on plastics. Most likely the community composition of plastics biofilm is shaped by an interaction between indirect effects of solar radiation on plastics and organic matter and direct effects on biofilm associated microbes.

## Conclusion

Based on our data, we conclude that there is no specific plastic associated bacterial community, recently coined “plastisphere”, but rather there is a part of the bacterial community, which is influenced by the characteristics of the individual plastic polymers and potentially their additives. This plastic type-specific bacterial community might be more abundant, as we found on the PVCs or less abundant, as in LDPE, HDPE and PP, probably depending, besides the composition of the additives, on other physico-chemical characteristics and environmental factors. Furthermore, differences in the composition of bacterial communities associated with different plastic surfaces appear to be higher in the initial than in later successional stages of biofilm formation. In the later stages of the biofilm formation these differences were more pronounced when samples where kept under dim light than under ambient light conditions. Taken together, this study reveals different colonization patterns by the microbial community depending on plastic-related properties, exposure to solar radiation, and most likely its additives.

## Supporting information

S1 File Additive measurements protocol. (PDF)

S2 File DNA extraction protocol. (PDF)

S3 File **SEM images of: (A) glass, (B) PP, (C) HDPE, (D) LDPE, (E) PVC DINP and (F) PVC DEHP incubated under both ambient and dim light conditions for one week, one month and two months.** The PVC DEHP sample for SEM analysis was lost during the incubation. Magnification is 450x.
(PDF)

S4 File Results of the post-hoc Tukey tests performed on the ANOVA comparing Shannon indexes (S1 Table). * indicates significant differences (p<0.05) between Shannon diversity indexes of the bacterial communities colonizing the different surfaces and the ambient water after (A) one week incubation, (B) one month of incubation, (C) two months of incubation. Diff–difference in means; lwr and upr–lower and upper confidence levels, respectively; p adj–adjusted p value for each pair.
(PDF)

S5 File Results of the post-hoc Tukey tests performed on the ANOVA comparing Simpson indexes (S1 Table). * indicates significant differences (p<0.05) between Simpson diversity indexes of the bacterial communities colonizing the different surfaces and the ambient water after (A) one week incubation, (B) one month of incubation, (C) two months of incubation. Diff–difference in means; lwr and upr–lower and upper confidence levels, respectively; p adj–adjusted p value for each pair.
(PDF)

S6 File Results of the post-hoc Tukey tests performed on the ANOVA comparing OTU richness (S1 Table). * indicates significant differences (p<0.05) between the OTU richness of the bacterial communities colonizing the different surfaces and the ambient water after (A) one week incubation, (B) one month of incubation, (C) two months of incubation. Diff–difference in means; lwr and upr–lower and upper confidence levels, respectively; p adj–adjusted p value for each pair.
(PDF)

S7 File **Values of *i_F_* representing the discrimination of the most abundant bacterial families (with 5% or more relative abundance in at least one of the samples) for either one of the plastics or glass and under the two different light conditions after (A) one month and (B) two months incubation.** Bars indicate mean of the duplicates and the grey lines connect the two duplicate values. Positive and negative values indicate bacterial families discriminating for plastic and glass, respectively. Bacterial families are ordered from the left to right from the most to the least relative abundance when considering all samples of that treatment and time point.
(PDF)

S8 File **Representation of the bacterial taxa which discriminated for one of the surfaces over all the others (considering both light treatments) determined by the discriminant linear analysis (LEfSe) after (A) one month and (B) two months of incubation.** The results of the LEfSe analysis is given in S7 Table.
(PDF)

S1 Table Results of the ANOVA tests comparing diversity and richness indexes of the bacterial communities between the different sampling times (Month), different surfaces and water (Type) and the two exposure treatments (Exposure). * indicates significant differences at p<0.05.
(PDF)

S2 Table Results of the PERMANOVA comparing bacterial community composition between the different sampling times (Month), different surfaces (plastics + glass + water) (Type) and the two exposure treatments (Exposure), with and without autotrophs. * indicates significant differences at p<0.05.
(PDF)

S3 Table Results of the PERMANOVA comparing bacterial community composition between the different surfaces (plastics + glass) (Type) and the two light exposure treatments (Exposure) for after one week, one month and two months of incubation. The Jaccard index was used as the distance measurement.
(PDF)

S4 Table Results of the PERMANOVA comparing bacterial community composition between the different surfaces (plastics + glass) (Type) and the two light exposure treatments (Exposure) for after one week, one month and two months of incubation. Bray-Curtis dissimilarity was used as the distance measurement.
(PDF)

S5 Table Results of the LEfSe analysis identifying taxa (down to the family level) discriminating for either glass or either one of the plastics after one week of incubation. (XLSX)

S6 Table Results of the LEfSe analysis identifying taxa (down to the family level) discriminating for either glass or either one of the plastics after one month of incubation. (XLSX)

S7 Table Results of the LEfSe analysis identifying taxa (down to the family level) discriminating for either glass or either one of the plastics after two months incubation. (XLSX)

S8 Table Results of the LEfSe analysis for each incubation time. It shows all OTUs that significantly discriminated for a specific surface at each sampling point.
(XLSX)

S1 Fig Scheme of the structure used to incubate the plastics and glass. Orange dots = buoys, big grey dot = main float with anchor. The frame was linked to a main float and anchored at the seafloor. The floating frame oriented itself to the direction of the currents allowing water flowing through the tubes in the dim light treatment. All the samples in the dim light treatment were placed in the center of the tube to avoid ensure similar low-light conditions for all the samples.
(PDF)

S2 Fig Map of the location where the in situ incubation was carried out. It was deployed in the Northern Adriatic Sea about 500 m off the coast of Rovinj, Croatia; the location is marked by a red dot.
(PDF)

S3 Fig NMDS representation of the similarity between the bacterial community composition of the biofilm on different surfaces and seawater, when excluding the autotrophic component, i.e., Cyanobacteria. Each point represents one sample. The grey polygons connect samples from each time point subjected to the same light conditions from substrates that presented relatively similar bacterial community compositions throughout the experiment.
(PDF)

S4 Fig Stack plot representing the relative abundances of the OTUs representing > 5% of the total bacterial community in each sample. OTUs representing < 5% of the total bacterial community were grouped into the category “Others”. The taxonomic classification of OTUs was obtained using a 97% similarity of the partial 16S rRNA gene and the SILVA database.
(PDF)
