# Supplementary material for: The composition of bacterial communities associated with plastic biofilms differs between different polymers and stages of biofilm succession
Source: PLoS One. 2019 Jun 5;14(6):e0217165. doi: 10.1371/journal.pone.0217165 (PMC6550384; doi:10.1371/journal.pone.0217165)
Supplement: S1 File — (PDF) [file pone.0217165.s001.pdf]

Additive measurements protocol

To determine the concentration of DEHP and DINP in the plastics throughout the experiment, phthalate concentrations in the samples were measured for all plastic types kept under ambient light condition. The biofilm was gently removed from the plastics using a wet paper tissue and the pieces were weighed after drying. The PVC pellets were dissolved in 5 mL tetrahydrofuran, followed by the precipitation of the polymer by adding 10 mL of hexane. An aliquot of the supernatant was diluted 1:100 and 980 µL of this dilution were transferred into a GC sample vial and 20 µL internal standard (DEHP D4, 50 ng/µL) were added prior to GC-MS analysis. PE and PP plastics were extracted using 5 mL tetrahydrofuran in an ultrasonic bath for 12 h. After adding 10 mL of hexane, the samples were measured. Gas chromatography-mass spectrometry (GC-MS, Agilent 5975C inert GC/MSD) was used for the analysis of the plasticizers (column: HP5-MS, 60 m; oven program: 80°C held for 30 s, ramp to 300°C at a rate of 20°C/min, hold 10 min; inlet temperature: 300°C; transfer line: 280°C; carrier gas: He at 1 mL min^{-1}).
