# Supplementary material for: The composition of bacterial communities associated with plastic biofilms differs between different polymers and stages of biofilm succession
Source: PLoS One. 2019 Jun 5;14(6):e0217165. doi: 10.1371/journal.pone.0217165 (PMC6550384; doi:10.1371/journal.pone.0217165)
Supplement: S2 File — (PDF) [file pone.0217165.s002.pdf]

DNA extraction protocol

After thawing the LDPE, HDPE and PP samples they were cut into smaller pieces and put in 2mL Eppendorf vials. The glass and PVC spheres were also put into 2 mL Eppendorf vials. Samples were incubated in 500 µL of lysis solution (kit) and 10 µL lysozyme (1000 U mL^{-1}) at 37°C for 30 min. Afterwards, 5 µL proteinase k (kit) was added and mixed by inverting 25 times. About 0.325 g of beads were added per tube before bead beating two times for 45 s at a vertical speed of 4 m/s (FastPrep™, MP Biomedicals, USA). After incubating at 55°C for another 30 min, 4 µL RNAse (kit) was added and mixed by inverting 50 times. Following a 30 min incubation at 37°C and 5 min on ice, 250 µL of protein precipitation solution (kit) was added to each tube and vortexed at high speed for 20 s. After centrifuging the tubes for 3 min at 14,000 *g*, the supernatant was transferred to a fresh tube containing 750 µL 100% isopropanol and inverted 50 times. Following another centrifuging step at 14,000 *g* for 5 min, the supernatant was discarded and tubes were drained from remaining isopropanol. As a washing step, 750µL 70% ethanol was added and the tubes were inverted several times. After centrifugation for an additional 3 min, the supernatant was discarded again and tubes were drained and air-dried. The remaining DNA extract was re-suspended in 40µL hydration solution (kit) and incubated at 65°C for 45 min to completely dissolve. DNA extracts were stored frozen at -80°C for later analyses.
