# Supplementary material for: The composition of bacterial communities associated with plastic biofilms differs between different polymers and stages of biofilm succession
Source: PLoS One. 2019 Jun 5;14(6):e0217165. doi: 10.1371/journal.pone.0217165 (PMC6550384; doi:10.1371/journal.pone.0217165)
Supplement: S3 File — SEM images of: (A) glass, (B) PP, (C) HDPE, (D) LDPE, (E) PVC DINP and (F) PVC DEHP incubated under both ambient and dim light conditions for one week, one month and two months. The PVC DEHP sample for SEM analysis was lost during the incubation. Magnification is 450x. (PDF) [file pone.0217165.s003.pdf]

(A) Glass

	Ambient light	Dim light
After one week	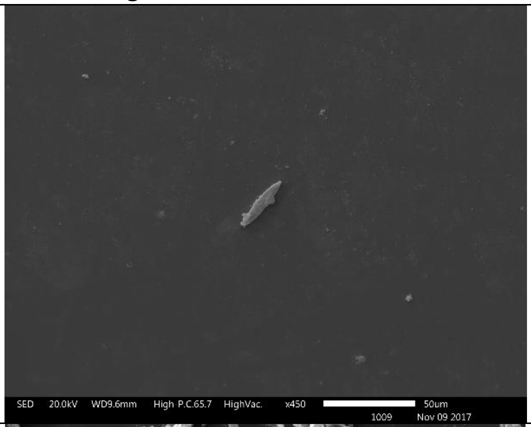	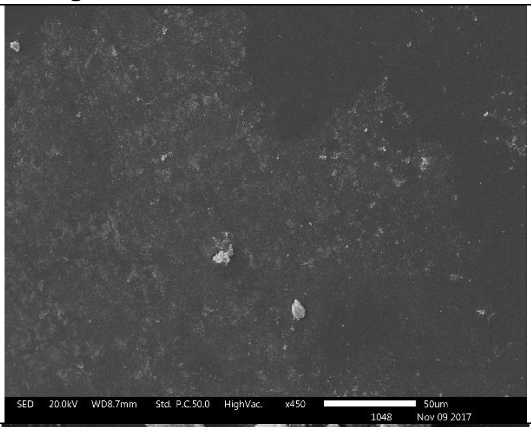
After one month	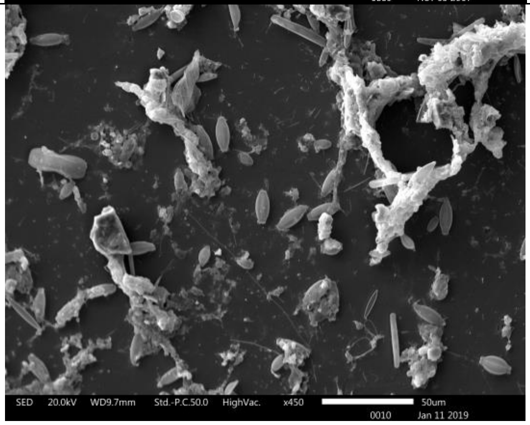	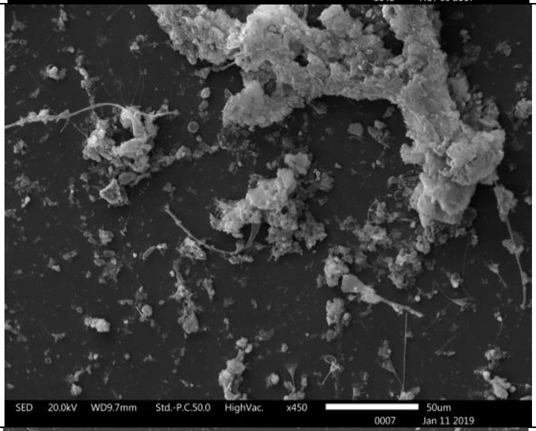
After two months	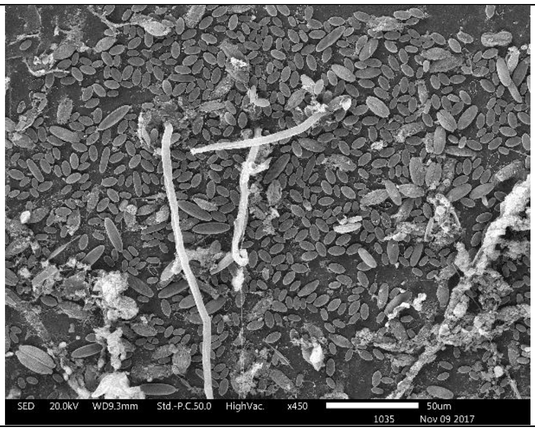	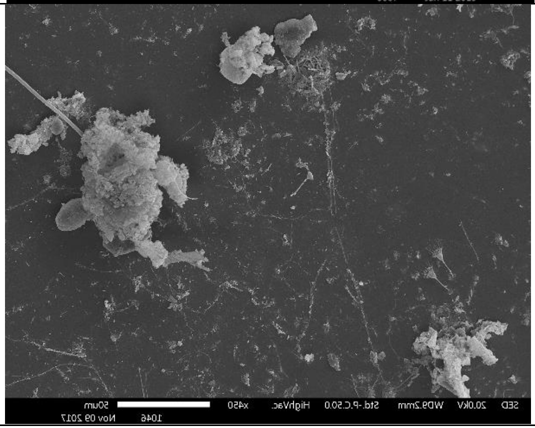

(B) LDPE

	Ambient light	Dim light
After one week	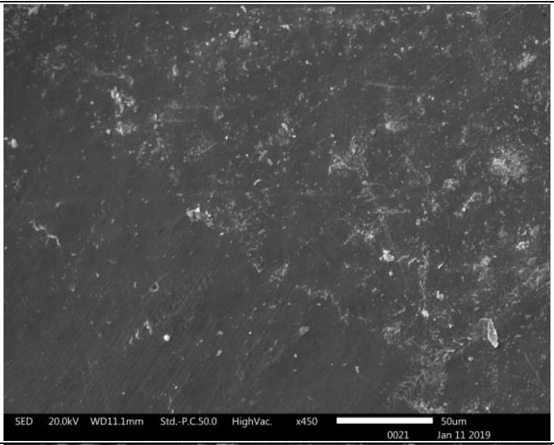	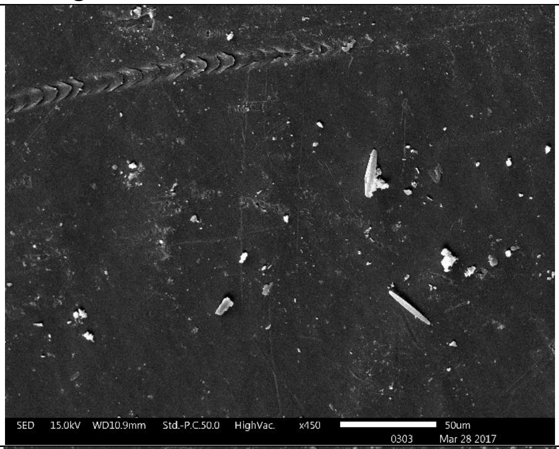
After one month	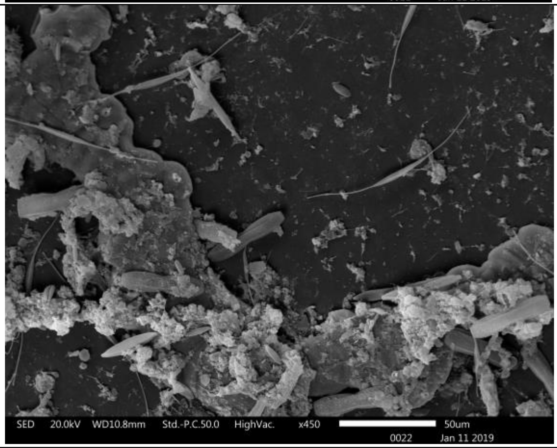	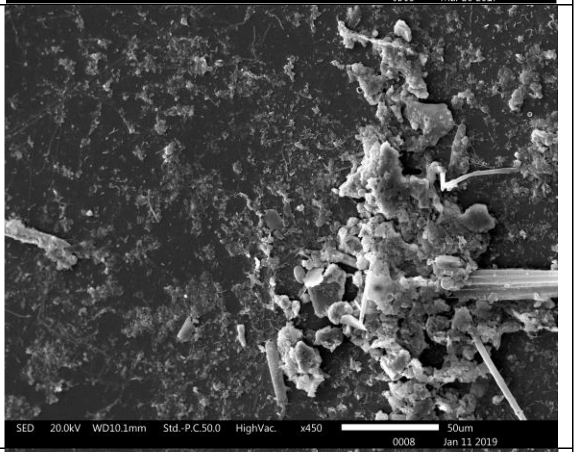
After two months	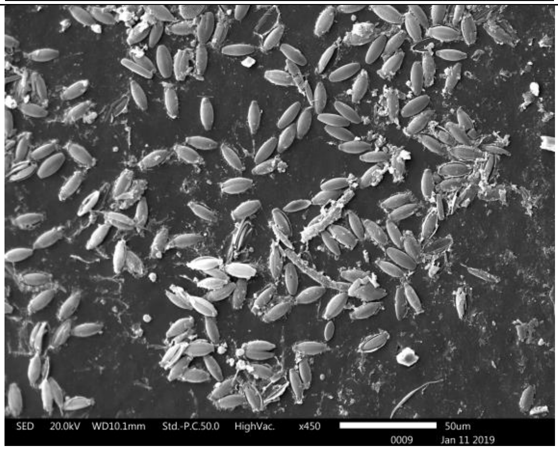	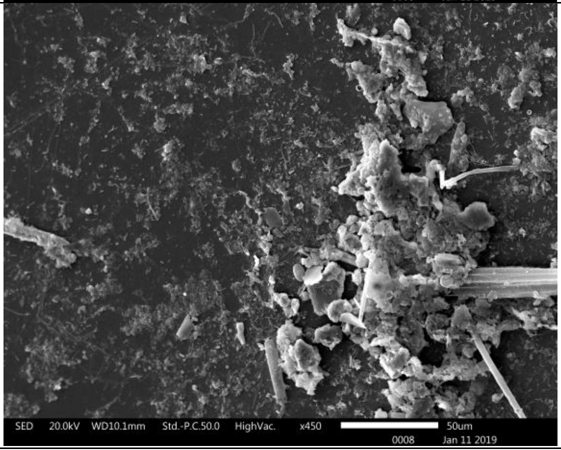

(C) HDPE

	Ambient light	Dim light
After one week	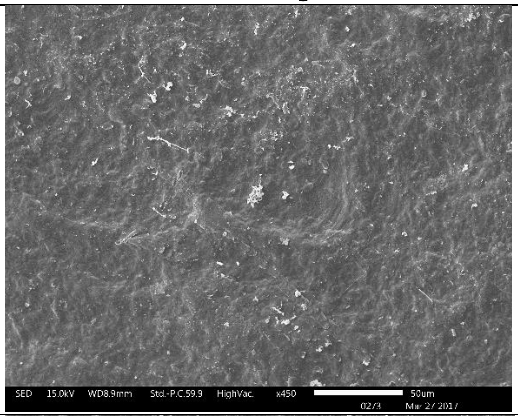	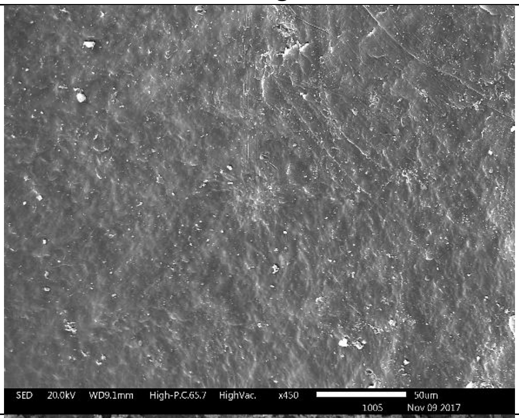
After one month	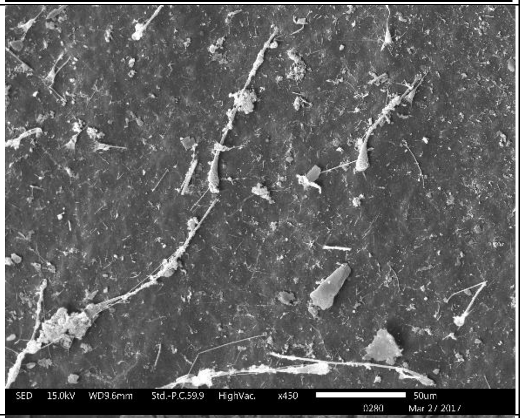	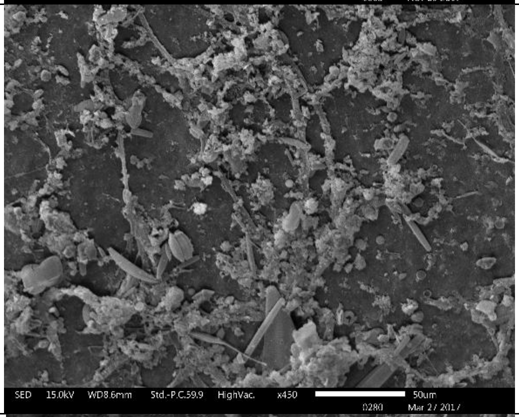
After two months	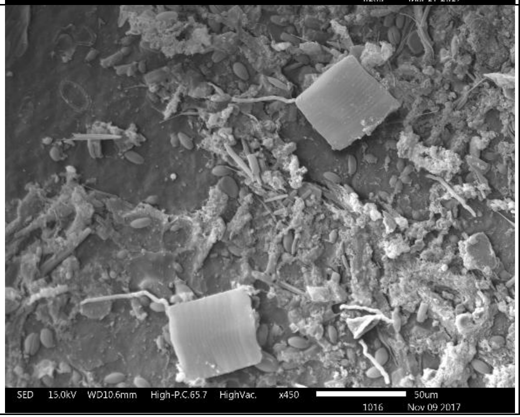	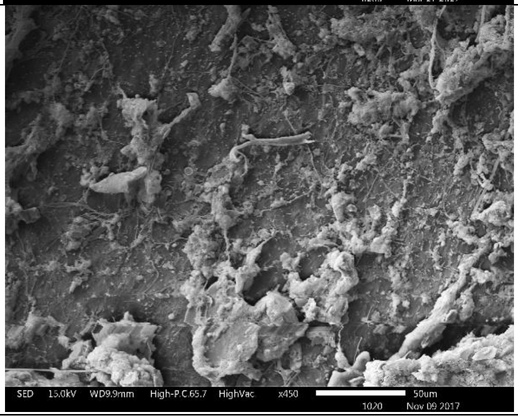

(D) PP

	Ambient light	Dim light
After one week	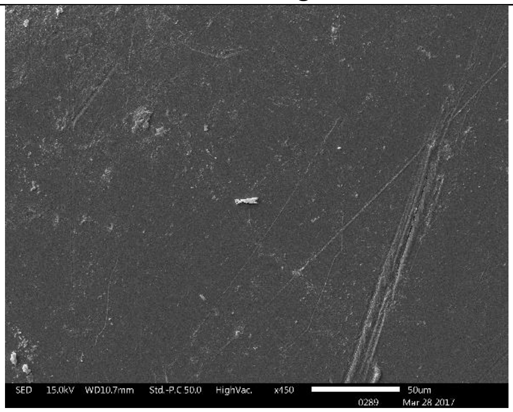	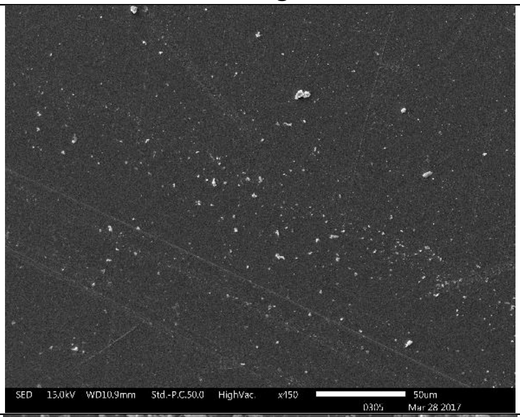
After one month	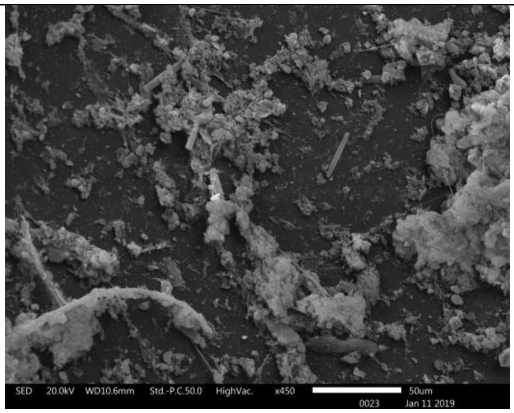	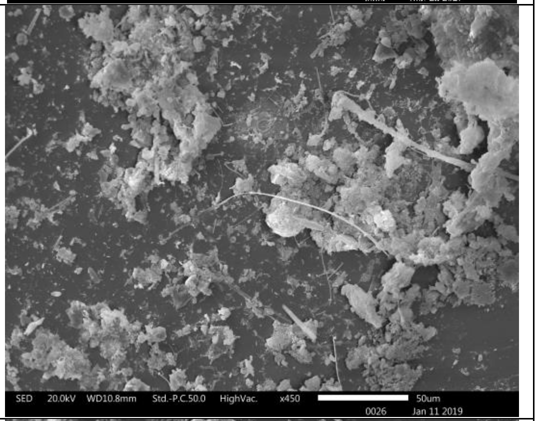
After two months	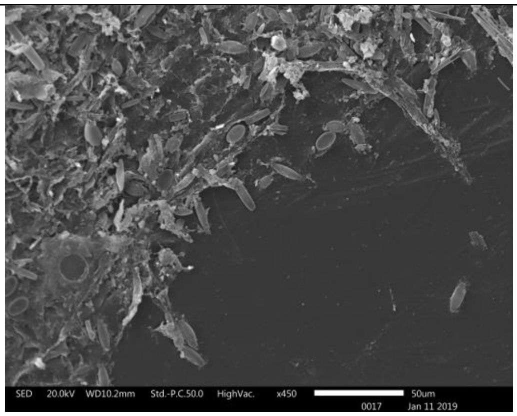	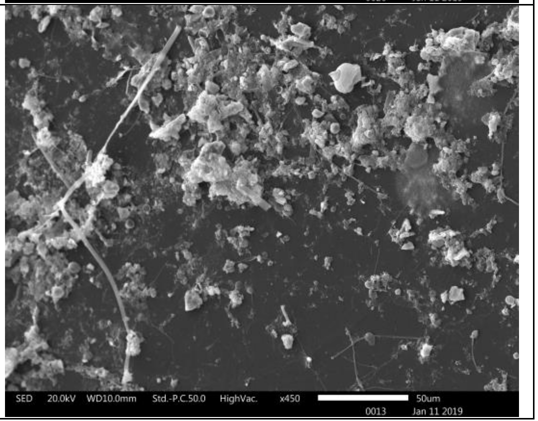

(E) PVC DEHP

	Ambient light	Dim light
After one week	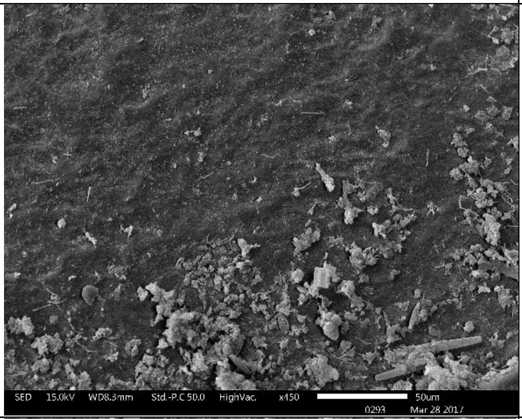	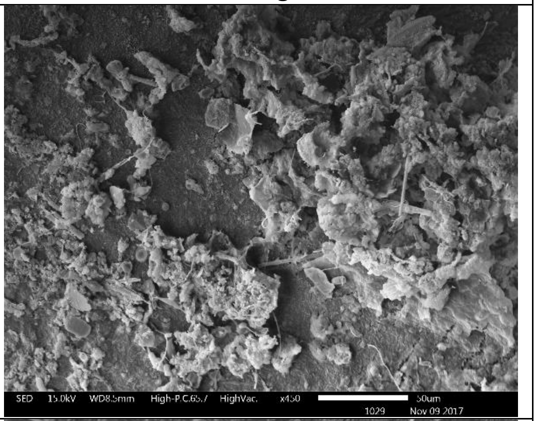
After one month	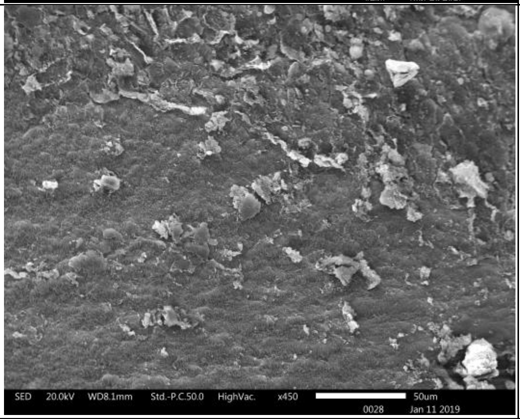	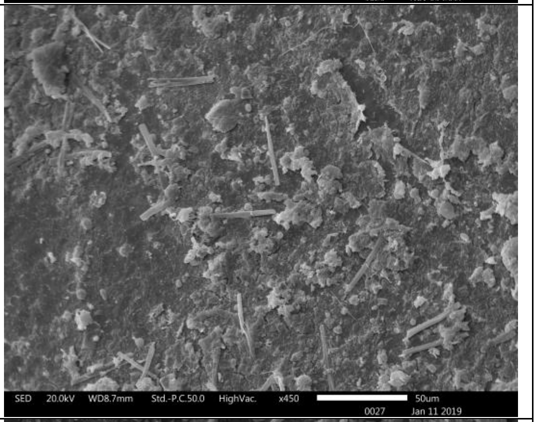
After two months	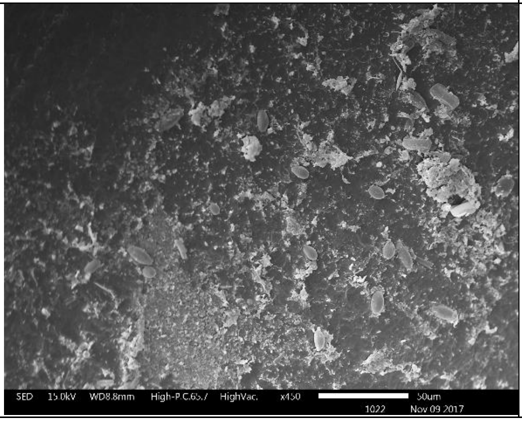	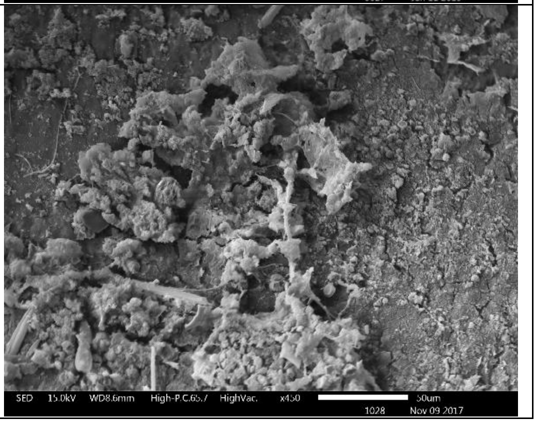

(F) PVC DEHP

	Ambient light	Dim light
After one week	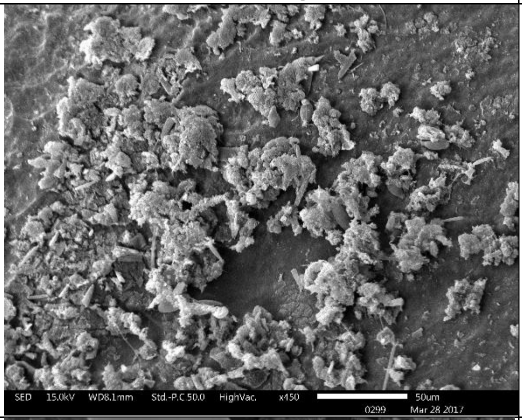	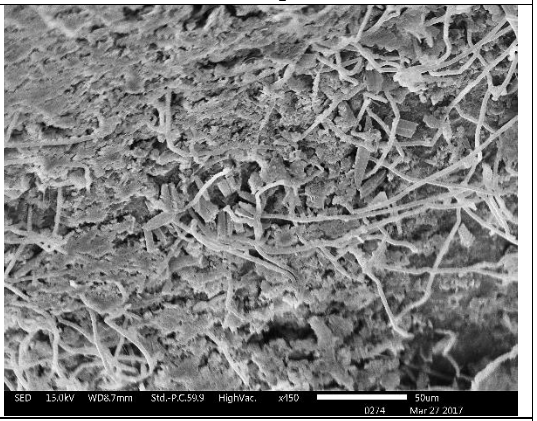
After one month	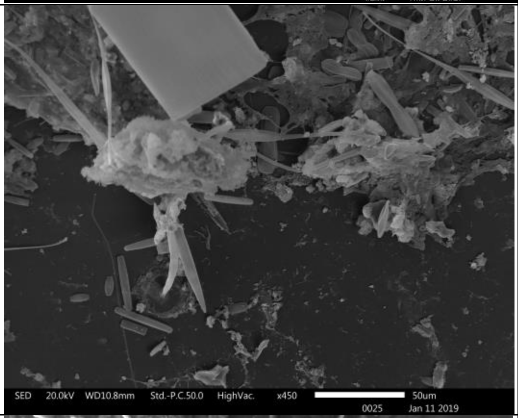	
After two months	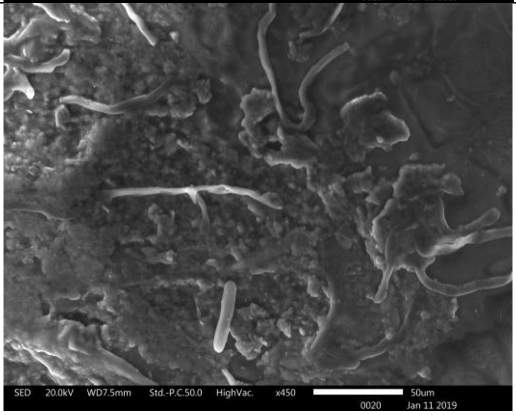	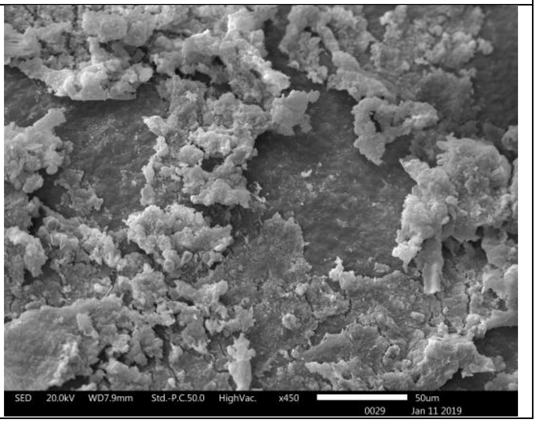
