# Supplementary material for: The composition of bacterial communities associated with plastic biofilms differs between different polymers and stages of biofilm succession
Source: PLoS One. 2019 Jun 5;14(6):e0217165. doi: 10.1371/journal.pone.0217165 (PMC6550384; doi:10.1371/journal.pone.0217165)
Supplement: S4 File — * indicates significant differences (p<0.05) between Shannon diversity indexes of the bacterial communities colonizing the different surfaces and the ambient water after (A) one week incubation, (B) one month of incubation, (C) two months of incubation. Diff–difference in means; lwr and upr–lower and upper confidence levels, respectively; p adj–adjusted p value for each pair. (PDF) [file pone.0217165.s004.pdf]

(A)

	1 week incubation							
	Dim light				Ambient light			
	diff	lwr	upr	p adj	diff	lwr	upr	p adj
**HDPE-Glass**	-0.05	-0.82	0.72	1.00	0.07	-0.69	0.84	1.00
**LDPE-Glass**	-0.29	-1.06	0.48	0.96	0.24	-0.53	1.00	1.00
**PP-Glass**	0.24	-0.53	1.00	1.00	0.00	-0.77	0.77	1.00
**PVC DEHP-Glass**	-0.70	-1.47	0.07	0.09	-0.13	-0.90	0.64	1.00
**PVC DINP-Glass**	-0.63	-1.40	0.14	0.16	-0.08	-0.84	0.69	1.00
**SW-Glass**	-0.29	-1.06	0.47	0.96	0.48	-0.29	1.24	0.51
**LDPE-HDPE**	-0.24	-1.01	0.53	0.99	0.16	-0.61	0.93	1.00
**PP-HDPE**	0.29	-0.48	1.06	0.97	-0.08	-0.85	0.69	1.00
**PVC DEHP-HDPE**	-0.65	-1.42	0.12	0.14	-0.20	-0.97	0.57	1.00
**PVC DINP-HDPE**	-0.58	-1.35	0.19	0.25	-0.15	-0.92	0.62	1.00
**SW-HDPE**	-0.24	-1.01	0.53	0.99	0.40	-0.37	1.17	0.74
**PP-LDPE**	0.53	-0.24	1.30	0.36	-0.24	-1.01	0.53	0.99
**PVC DEHP-LDPE**	-0.41	-1.18	0.36	0.71	-0.36	-1.13	0.40	0.84
**PVC DINP-LDPE**	-0.34	-1.11	0.43	0.90	-0.31	-1.08	0.46	0.94
**SW-LDPE**	0.00	-0.77	0.77	1.00	0.24	-0.53	1.01	0.99
**PVC DEHP-PP**	-0.94	-1.71	-0.17	**0.01***	-0.13	-0.89	0.64	1.00
**PVC DINP-PP**	-0.87	-1.64	-0.10	**0.02***	-0.07	-0.84	0.70	1.00
**SW-PP**	-0.53	-1.30	0.24	0.36	0.48	-0.29	1.25	0.50
**PVC DINP-PVC DEHP**	0.07	-0.70	0.84	1.00	0.05	-0.72	0.82	1.00
**SW-PVC DEHP**	0.41	-0.36	1.18	0.72	0.60	-0.16	1.37	0.20
**SW-PVC DINP**	0.34	-0.43	1.11	0.90	0.55	-0.22	1.32	0.31

(B)

	1 month incubation							
	Dim light				Ambient light			
	diff	lwr	upr	p adj	diff	lwr	upr	p adj
**HDPE-Glass**	-0.37	-1.16	0.42	0.79	0.28	-0.51	1.07	0.96
**LDPE-Glass**	-0.09	-0.73	0.56	1.00	-0.14	-0.78	0.50	1.00
**PP-Glass**	0.43	-0.22	1.07	0.36	0.79	0.15	1.43	**0.01***
**PVC DEHP-Glass**	-1.48	-2.12	-0.84	**<0.01***	-0.08	-0.72	0.56	1.00
**PVC DINP-Glass**	-1.14	-1.92	-0.35	**<0.01***	0.39	-0.25	1.03	0.46
**SW-Glass**	-1.40	-2.04	-0.76	**<0.01***	-0.31	-0.95	0.33	0.75
**LDPE-HDPE**	0.28	-0.50	1.07	0.96	-0.42	-1.21	0.37	0.63
**PP-HDPE**	0.80	0.01	1.58	**0.04***	0.51	-0.28	1.29	0.39
**PVC DEHP-HDPE**	-1.11	-1.90	-0.32	**<0.01***	-0.36	-1.15	0.43	0.82
**PVC DINP-HDPE**	-0.77	-1.68	0.14	0.13	0.11	-0.68	0.90	1.00
**SW-HDPE**	-1.03	-1.82	-0.25	**0.01***	-0.59	-1.38	0.20	0.22
**PP-LDPE**	0.51	-0.13	1.15	0.17	0.93	0.29	1.57	**<0.01***
**PVC DEHP-LDPE**	-1.39	-2.04	-0.75	**<0.01***	0.06	-0.58	0.71	1.00
**PVC DINP-LDPE**	-1.05	-1.84	-0.26	**0.01***	0.53	-0.11	1.18	0.14
**SW-LDPE**	-1.32	-1.96	-0.67	**<0.01***	-0.17	-0.81	0.47	1.00
**PVC DEHP-PP**	-1.91	-2.55	-1.26	**<0.01***	-0.87	-1.51	-0.22	**0.01***
**PVC DINP-PP**	-1.56	-2.35	-0.78	**<0.01***	-0.40	-1.04	0.25	0.45
**SW-PP**	-1.83	-2.47	-1.19	**<0.01***	-1.10	-1.74	-0.46	**<0.01***
**PVC DINP-PVC DEHP**	0.34	-0.44	1.13	0.86	0.47	-0.17	1.11	0.25
**SW-PVC DEHP**	0.08	-0.56	0.72	1.00	-0.23	-0.88	0.41	0.96
**SW-PVC DINP**	-0.26	-1.05	0.52	0.98	-0.70	-1.35	-0.06	0.03

(C)

	2 months incubation							
	Dim light				Ambient light			
	**diff**	**lwr**	**upr**	**p adj**	**diff**	**lwr**	**upr**	**p adj**
**HDPE-Glass**	0.74	-0.98	2.45	0.86	-0.52	-2.24	1.19	0.99
**LDPE-Glass**	0.66	-1.44	2.76	0.99	-0.40	-2.11	1.31	1.00
**PP-Glass**	-0.01	-1.72	1.71	1.00	-0.17	-1.88	1.54	1.00
**PVC DEHP-Glass**	-1.11	-2.82	0.61	0.39	-0.70	-2.42	1.01	0.90
**PVC DINP-Glass**	-0.60	-2.70	1.50	0.99	-0.65	-2.36	1.07	0.94
**SW-Glass**	-1.16	-3.26	0.94	0.59	-0.64	-2.74	1.46	0.99
**LDPE-HDPE**	-0.08	-2.18	2.02	1.00	0.12	-1.59	1.84	1.00
**PP-HDPE**	-0.74	-2.46	0.97	0.86	0.36	-1.36	2.07	1.00
**PVC DEHP-HDPE**	-1.84	-3.56	-0.13	**0.03***	-0.18	-1.89	1.53	1.00
**PVC DINP-HDPE**	-1.34	-3.44	0.76	0.40	-0.12	-1.84	1.59	1.00
**SW-HDPE**	-1.90	-4.00	0.20	0.09	-0.12	-2.22	1.98	1.00
**PP-LDPE**	-0.66	-2.76	1.44	0.99	0.23	-1.48	1.95	1.00
**PVC DEHP-LDPE**	-1.76	-3.86	0.34	0.13	-0.30	-2.02	1.41	1.00
**PVC DINP-LDPE**	-1.26	-3.69	1.16	0.67	-0.25	-1.96	1.47	1.00
**SW-LDPE**	-1.82	-4.24	0.60	0.22	-0.24	-2.34	1.86	1.00
**PVC DEHP-PP**	-1.10	-2.81	0.61	0.40	-0.53	-2.25	1.18	0.99
**PVC DINP-PP**	-0.60	-2.70	1.50	0.99	-0.48	-2.19	1.24	1.00
**SW-PP**	-1.16	-3.25	0.94	0.60	-0.47	-2.57	1.63	1.00
**PVC DINP-PVC DEHP**	0.50	-1.60	2.60	1.00	0.06	-1.66	1.77	1.00
**SW-PVC DEHP**	-0.06	-2.15	2.04	1.00	0.06	-2.04	2.16	1.00
**SW-PVC DINP**	-0.56	-2.98	1.87	1.00	0.01	-2.09	2.11	1.00
