# Supplementary material for: The composition of bacterial communities associated with plastic biofilms differs between different polymers and stages of biofilm succession
Source: PLoS One. 2019 Jun 5;14(6):e0217165. doi: 10.1371/journal.pone.0217165 (PMC6550384; doi:10.1371/journal.pone.0217165)
Supplement: S6 File — * indicates significant differences (p<0.05) between the OTU richness of the bacterial communities colonizing the different surfaces and the ambient water after (A) one week incubation, (B) one month of incubation, (C) two months of incubation. Diff–difference in means; lwr and upr–lower and upper confidence levels, respectively; p adj–adjusted p value for each pair. (PDF) [file pone.0217165.s006.pdf]

**(A)**

| | 1 week incubation | | | | | | | |
|---|---|---|---|---|---|---|---|---|
| | Dim light | | | | Ambient light | | | |
| | diff | lwr | upr | p adj | diff | lwr | upr | p adj |
| HDPE-Glass | -32.5 | -188 | 123 | 1.00 | -31.8 | -187 | 124 | 1.00 |
| LDPE-Glass | -20.4 | -17 | 135 | 1.00 | -29.9 | -185 | 125 | 1.00 |
| PP-Glass | -24.3 | -180 | 131 | 1.00 | -25.9 | -181 | 129 | 1.00 |
| PVC DEHP-Glass | -120 | -276 | 35.4 | 0.22 | -107 | -263 | 48.7 | 0.36 |
| PVC DINP-Glass | -105 | -261 | 50.7 | 0.39 | -83.3 | -239 | 72.5 | 0.71 |
| SW-Glass | -40.2 | -196 | 115 | 1.00 | 24.3 | -131 | 180 | 1.00 |
| LDPE-HDPE | 12.1 | -143 | 168 | 1.00 | 1.98 | -153 | 157 | 1.00 |
| PP-HDPE | 8.21 | -147 | 164 | 1.00 | 5.97 | -149 | 161 | 1.00 |
| PVC DEHP-HDPE | -87.8 | -243 | 68 | 0.64 | -75.2 | -231 | 80.6 | 0.82 |
| PVC DINP-HDPE | -72.6 | -228 | 83.2 | 0.86 | -51.4 | -207 | 104 | 0.99 |
| SW-HDPE | -7.69 | -163 | 148 | 1.00 | 56.2 | -99.6 | 212 | 0.98 |
| PP-LDPE | -3.91 | -159 | 151 | 1.00 | 3.99 | -151 | 159 | 1.00 |
| PVC DEHP-LDPE | -100 | -255 | 55.8 | 0.46 | -77.2 | -233 | 78.6 | 0.80 |
| PVC DINP-LDPE | -84.7 | -240 | 71.1 | 0.69 | -53.4 | -209 | 102 | 0.99 |
| SW-LDPE | -19.8 | -175 | 136 | 1.00 | 54.2 | -101 | 210 | 0.98 |
| PVC DEHP-PP | -96.1 | -251 | 59.7 | 0.52 | -81.2 | -237 | 74.6 | 0.74 |
| PVC DINP-PP | -80.8 | -236 | 75.0 | 0.75 | -57.4 | -213 | 98.4 | 0.97 |
| SW-PP | -15.8 | -171 | 140 | 1.00 | 50.2 | -105 | 206 | 0.99 |
| PVC DINP-PVC DEHP | 15.2 | -140 | 171 | 1.00 | 23.8 | -132 | 179 | 1.00 |
| SW-PVC DEHP | 80.2 | -75.6 | 236 | 0.76 | 131 | -24.3 | 287 | 0.14 |
| SW-PVC DINP | 64.9 | -90.9 | 220 | 0.93 | 107 | -48.2 | 263 | 0.36 |

**(B)**

| | 1 month incubation | | | | | | | |
|---|---|---|---|---|---|---|---|---|
| | Dim light | | | | Ambient light | | | |
| | diff | lwr | Upr | p adj | diff | lwr | upr | p adj |
| **HDPE-Glass** | 28.3 | -194 | 251 | 1.00 | -40.9 | -263 | 181.68 | 1.00 |
| **LDPE-Glass** | 51.2 | -130 | 233 | 1.00 | -135 | -317 | 46.18 | 0.23 |
| **PP-Glass** | 54.7 | -127 | 236 | 0.99 | 44.5 | -137 | 226.36 | 1.00 |
| **PVC DEHP-Glass** | -318 | -500 | -136 | **<0.01*** | -114 | -296 | 67.34 | 0.42 |
| **PVC DINP-Glass** | -191 | -413 | 31.6 | 0.12 | -75.4 | -257 | 106.40 | 0.89 |
| **SW-Glass** | -356 | -538 | -175 | **<0.01*** | -264 | -446 | -82.52 | **<0.01*** |
| **LDPE-HDPE** | 22.8 | -199 | 245 | 1.00 | -94.6 | -317 | 128.03 | 0.87 |
| **PP-HDPE** | 26.4 | -196 | 249 | 1.00 | 85.5 | -137 | 308.22 | 0.93 |
| **PVC DEHP-HDPE** | -346 | -569 | -124 | **<0.01*** | -73.4 | -296 | 149.19 | 0.98 |
| **PVC DINP-HDPE** | -219 | -476 | 37.7 | 0.12 | -34.4 | -257 | 188.25 | 1.00 |
| **SW-HDPE** | -385 | -608 | -162 | **<0.01*** | -223 | -446 | -0.67 | **0.05** |
| **PP-LDPE** | 3.57 | -178 | 185 | 1.00 | 180 | -1.63 | 361.99 | 0.05 |
| **PVC DEHP-LDPE** | -369 | -551 | -187 | **<0.01*** | 21.1 | -160 | 202.97 | 1.00 |
| **PVC DINP-LDPE** | -242 | -464 | -19.5 | **0.03** | 60.2 | -121 | 242.03 | 0.98 |
| **SW-LDPE** | -408 | -590 | -226 | **<0.01*** | -128 | -310 | 53.11 | 0.28 |
| **PVC DEHP-PP** | -373 | -555 | -191 | **<0.01*** | -159 | -340 | 22.79 | 0.11 |
| **PVC DINP-PP** | -245 | -468 | -23.1 | **0.03** | -119 | -301 | 61.85 | 0.36 |
| **SW-PP** | -411 | -593 | -229 | **<0.01*** | -308 | -490 | -127.0 | **<0.01*** |
| **PVC DINP-PVC DEHP** | 127 | -95.2 | 350 | 0.55 | 39.0 | -142 | 220.87 | 1.00 |
| **SW-PVC DEHP** | -38.5 | -220 | 143 | 1.00 | -149 | -331 | 31.96 | 0.14 |
| **SW-PVC DINP** | -165 | -388 | 56.6 | 0.23 | -188 | -370 | -7.11 | **0.04** |

**(C)**

| | 2 months incubation | | | | | | | |
|---|---|---|---|---|---|---|---|---|
| | Dim light | | | | Ambient light | | | |
| | diff | lwr | upr | p adj | diff | lwr | upr | p adj |
| **HDPE-Glass** | 113 | -77.7 | 304 | 0.23 | -125 | -481 | 230 | 0.75 |
| **LDPE-Glass** | 63.1 | -170 | 296 | 0.81 | -102 | -458 | 253 | 0.87 |
| **PP-Glass** | 41.9 | -148 | 232 | 0.90 | 0.84 | -355 | 356 | 1.00 |
| **PVC DEHP-Glass** | -157 | -348 | 32.9 | 0.09 | -175 | -531 | 180 | 0.47 |
| **PVC DINP-Glass** | -46.8 | -280 | 186 | 0.93 | -170 | -526 | 185 | 0.50 |
| **SW-Glass** | -226 | -460 | 7.04 | 0.06 | -258 | -694 | 177 | 0.31 |
| **LDPE-HDPE** | -50.0 | -283 | 183 | 0.91 | 23.2 | -332 | 379 | 1.00 |
| **PP-HDPE** | -71.1 | -262 | 119 | 0.58 | 126 | -229 | 482 | 0.75 |
| **PVC DEHP-HDPE** | -271 | -462 | -80.2 | **0.01*** | -50.00 | -405 | 305 | 0.99 |
| **PVC DINP-HDPE** | -160 | -393 | 73.7 | 0.16 | -44.2 | -400 | 311 | 1.00 |
| **SW-HDPE** | -339 | -573 | -106 | **0.01*** | -132 | -568 | 303 | 0.84 |
| **PP-LDPE** | -21.1 | -254 | 212 | 1.00 | 103 | -252 | 459 | 0.87 |
| **PVC DEHP-LDPE** | -221 | -454 | 12.7 | 0.06 | -73.2 | -429 | 282 | 0.97 |
| **PVC DINP-LDPE** | -110 | -379 | 159 | 0.51 | -67.4 | -423 | 288 | 0.98 |
| **SW-LDPE** | -289 | -559 | -19.9 | **0.04*** | -155 | -591 | 280 | 0.74 |
| **PVC DEHP-PP** | -199 | -390 | -9.02 | **0.04*** | -176 | -532 | 179 | 0.46 |
| **PVC DINP-PP** | -88.8 | -322 | 144 | 0.56 | -171 | -526 | 184 | 0.49 |
| **SW-PP** | -268 | -502 | -34.9 | **0.03*** | -259 | -695 | 176 | 0.30 |
| **PVC DINP-PVC DEHP** | 111 | -122 | 344 | 0.38 | 5.78 | -350 | 361 | 1.00 |
| **SW-PVC DEHP** | -68.7 | -302 | 165 | 0.75 | -82.7 | -518 | 353 | 0.98 |
| **SW-PVC DINP** | -179 | -449 | 90.1 | 0.17 | -88.5 | -524 | 347 | 0.97 |
