# Supplementary figures and images for: The composition of bacterial communities associated with plastic biofilms differs between different polymers and stages of biofilm succession
Source: PLoS One. 2019 Jun 5;14(6):e0217165. doi: 10.1371/journal.pone.0217165 (PMC6550384; doi:10.1371/journal.pone.0217165)

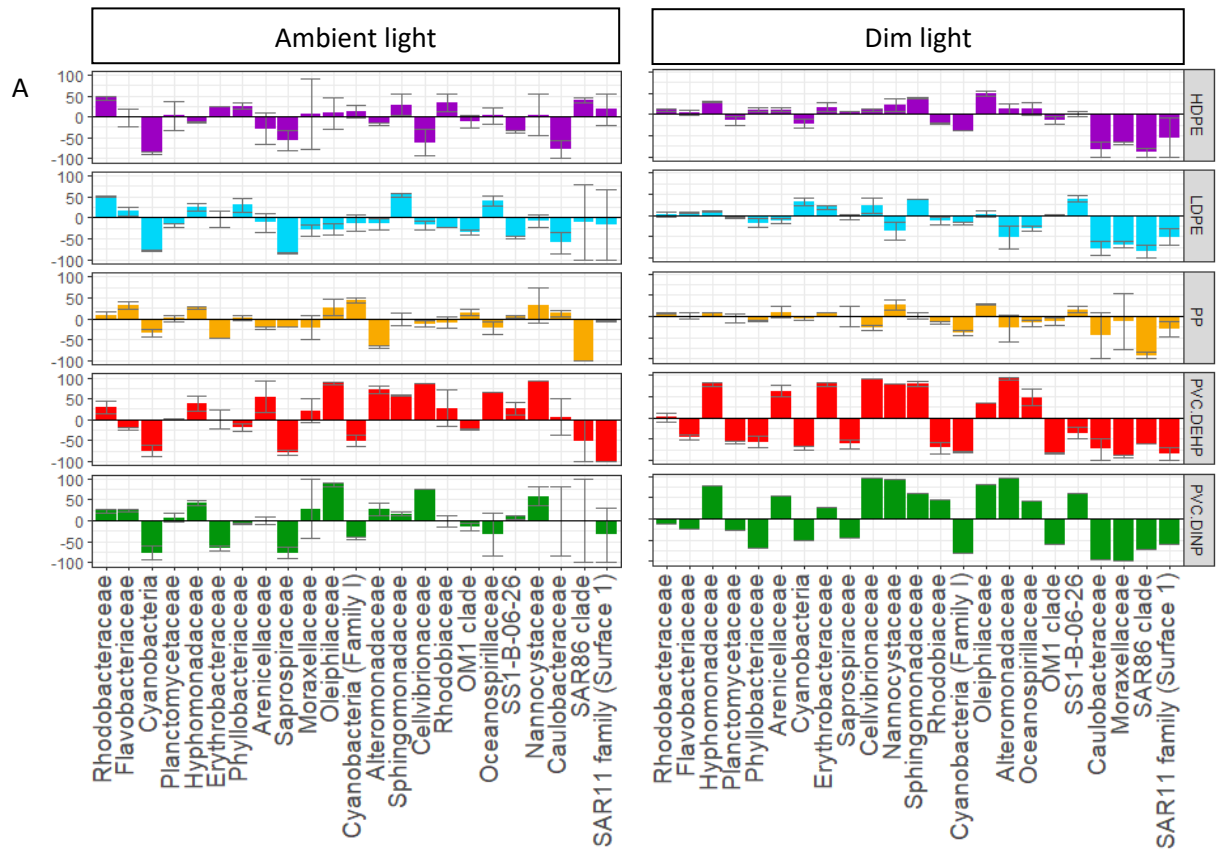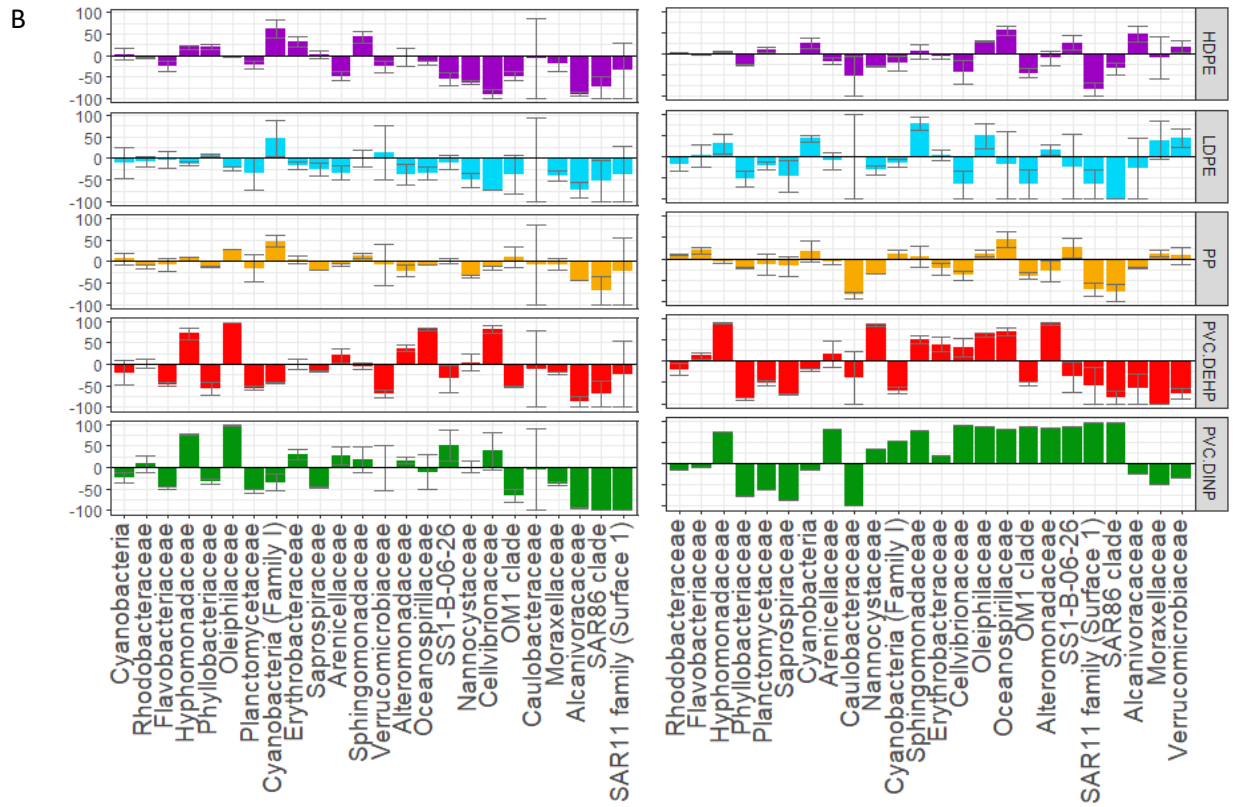

Supplement: S7 File — Values of iF representing the discrimination of the most abundant bacterial families (with 5% or more relative abundance in at least one of the samples) for either one of the plastics or glass and under the two different light conditions after (A) one month and (B) two months incubation. Bars indicate mean of the duplicates and the grey lines connect the two duplicate values. Positive and negative values indicate bacterial families discriminating for plastic and glass, respectively. Bacterial families are ordered from the left to right from the most to the least relative abundance when considering all samples of that treatment and time point. (PDF) [file pone.0217165.s007.pdf]

A

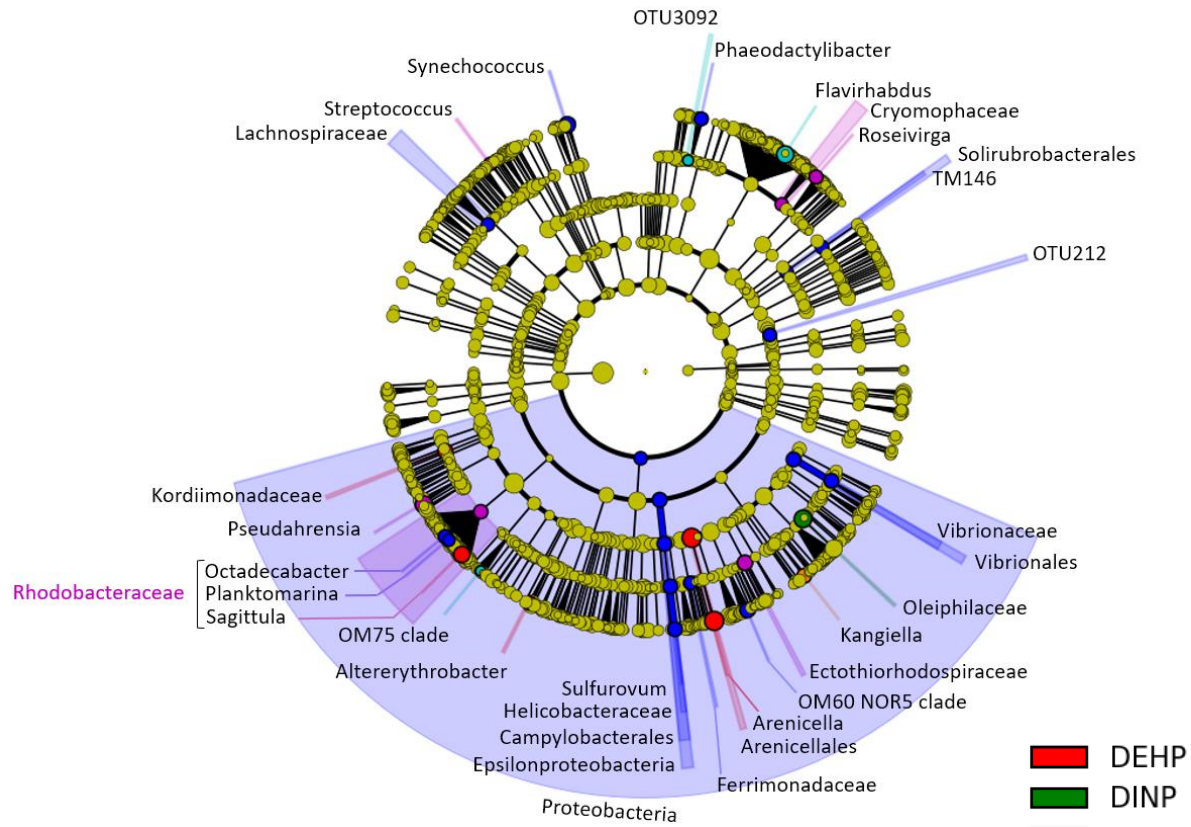

B

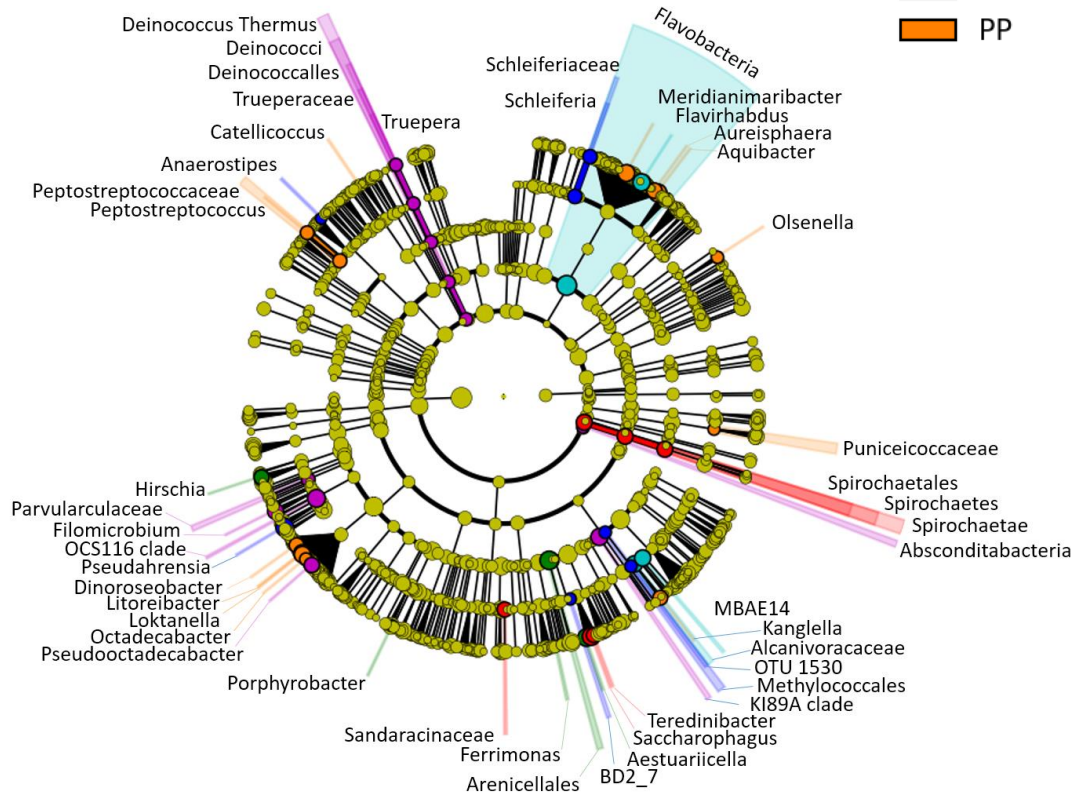

Supplement: S8 File — Representation of the bacterial taxa which discriminated for one of the surfaces over all the others (considering both light treatments) determined by the discriminant linear analysis (LEfSe) after (A) one month and (B) two months of incubation. The results of the LEfSe analysis is given in S7 Table. (PDF) [file pone.0217165.s008.pdf]

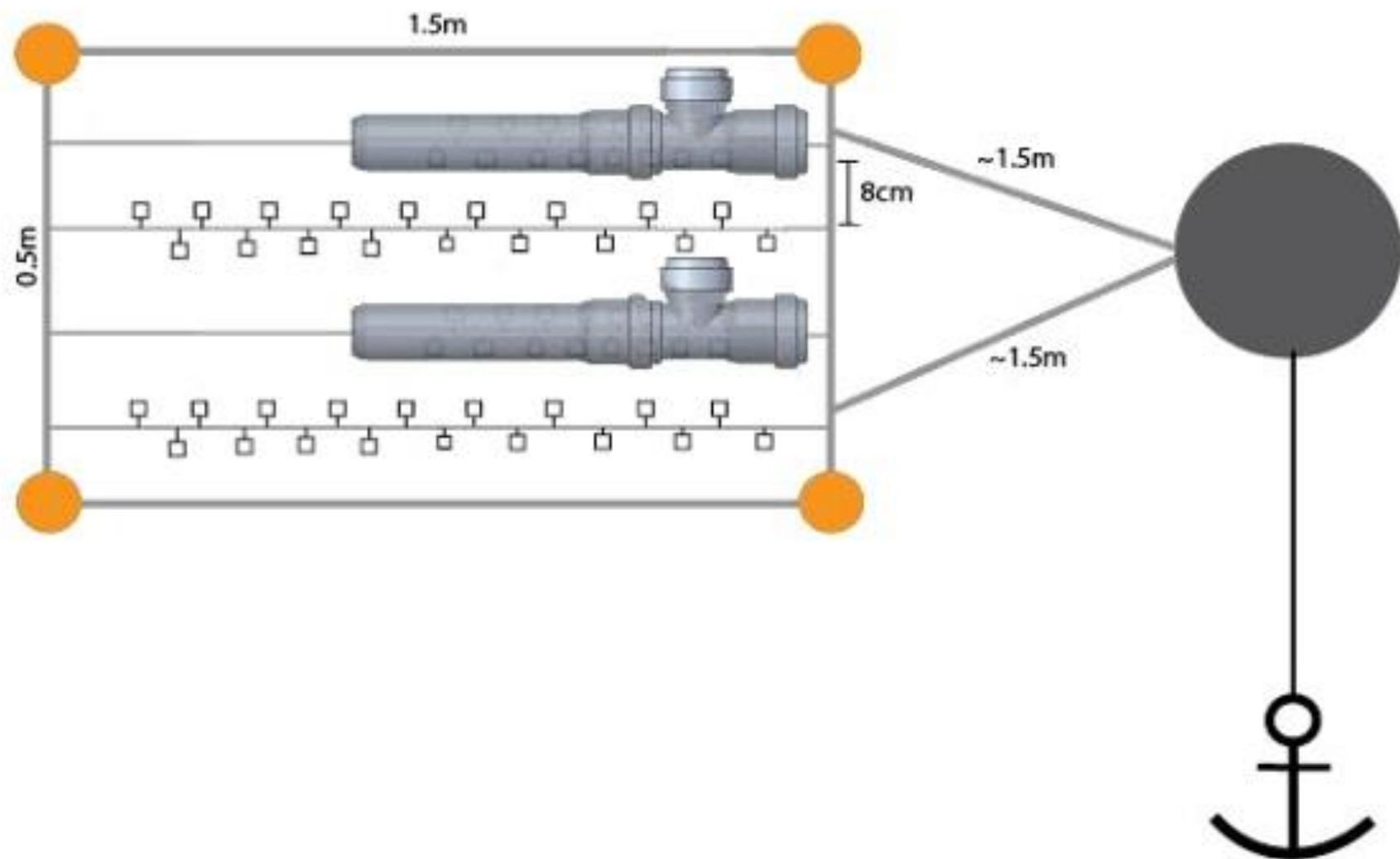

Supplement: S1 Fig — Orange dots = buoys, big grey dot = main float with anchor. The frame was linked to a main float and anchored at the seafloor. The floating frame oriented itself to the direction of the currents allowing water flowing through the tubes in the dim light treatment. All the samples in the dim light treatment were placed in the center of the tube to avoid ensure similar low-light conditions for all the samples. (PDF) [file pone.0217165.s017.pdf]

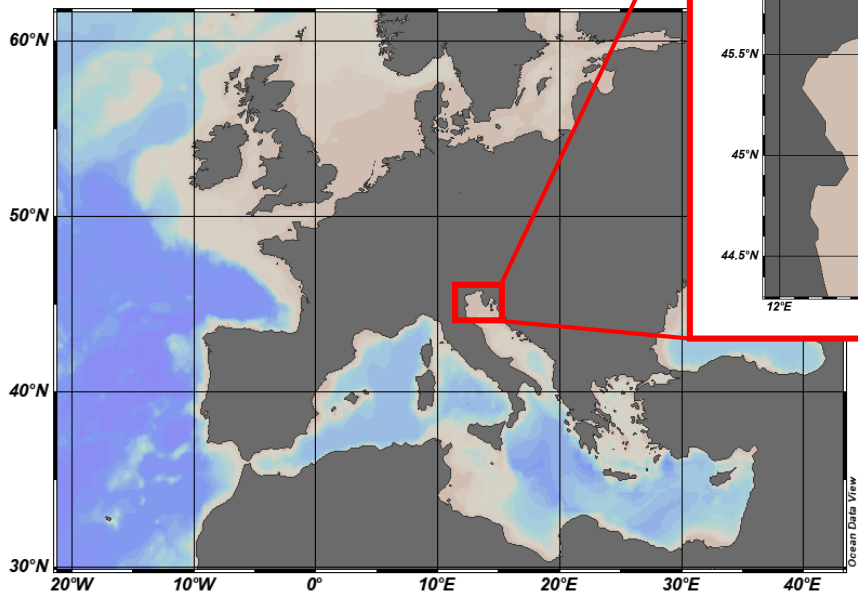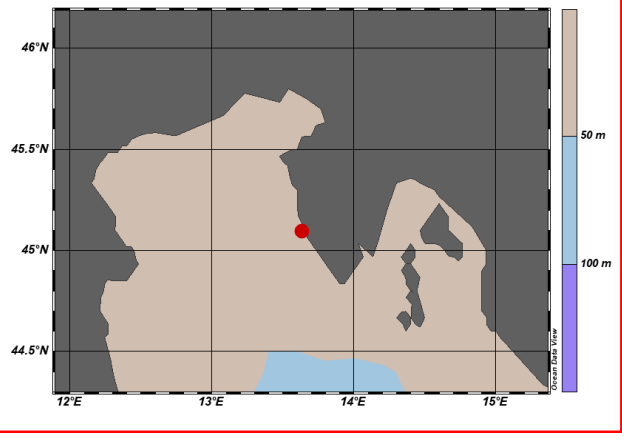

Supplement: S2 Fig — It was deployed in the Northern Adriatic Sea about 500 m off the coast of Rovinj, Croatia; the location is marked by a red dot. (PDF) [file pone.0217165.s018.pdf]

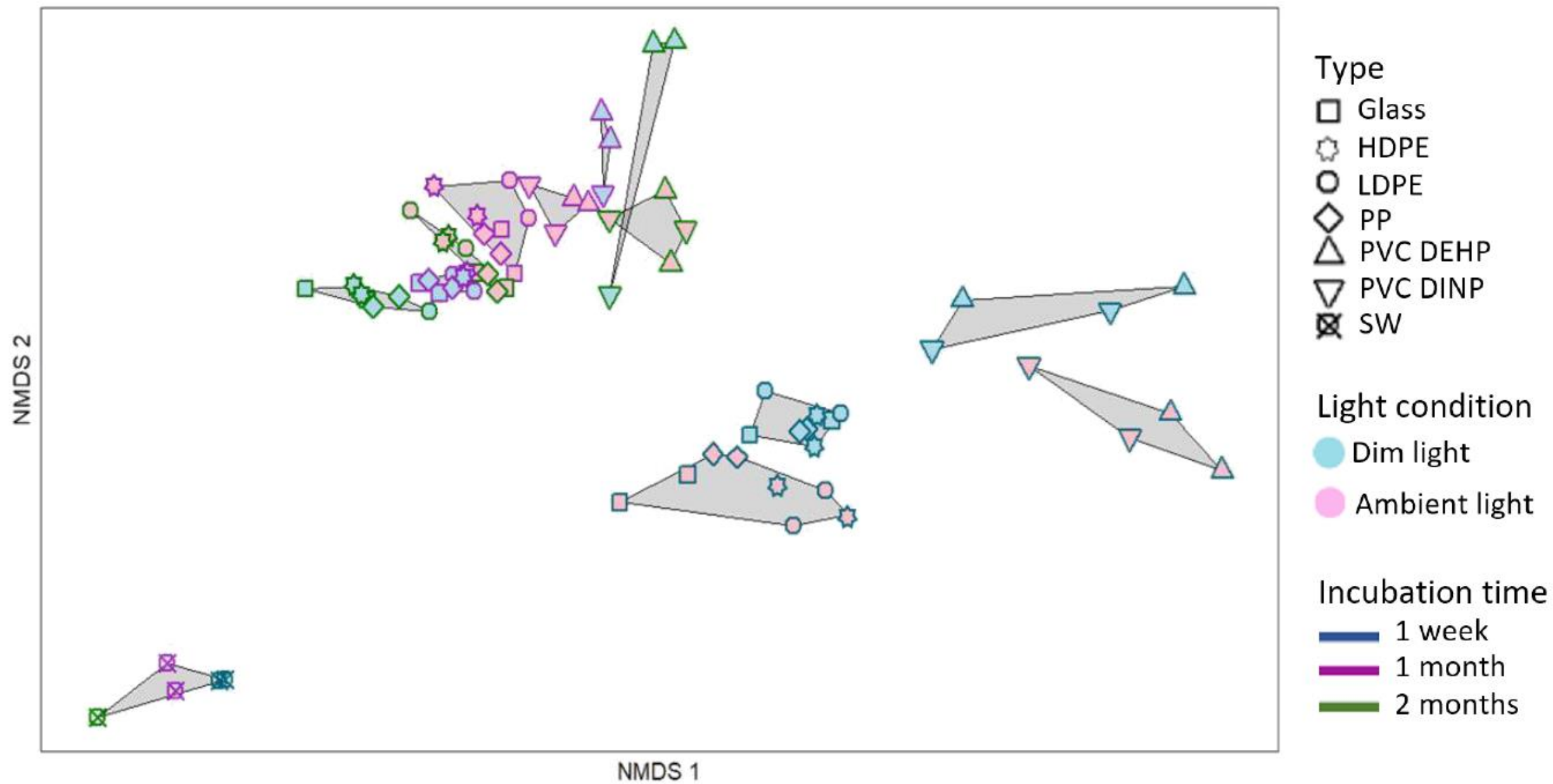

Supplement: S3 Fig — Each point represents one sample. The grey polygons connect samples from each time point subjected to the same light conditions from substrates that presented relatively similar bacterial community compositions throughout the experiment. (PDF) [file pone.0217165.s019.pdf]
