# Supplementary material for: The composition of bacterial communities associated with plastic biofilms differs between different polymers and stages of biofilm succession
Source: PLoS One. 2019 Jun 5;14(6):e0217165. doi: 10.1371/journal.pone.0217165 (PMC6550384; doi:10.1371/journal.pone.0217165)
Supplement: S1 Table — * indicates significant differences at p<0.05. (PDF) [file pone.0217165.s009.pdf]

	OTU richness		Shannon diversity		Simpson diversity	
	F value	Pr(>F)	F value	Pr(>F)	F value	Pr(>F)
Type	24.7	$6.8 x10^{-11}$*	20.6	$6.9 x10^{-10}$*	18	$3.6 x10^{-9}$*
Month	110	$2.3 x10^{-15}$*	96.3	$2.6 x10^{-11}$*	130	$3.5 x10^{-13}$*
Exposure	30.4	$4.1 x10^{-06}$*	67.2	$2.3 x10^{-12}$*	55	$3.3 x10^{-11}$*
Type: Month	4.0	$7.0 x10^{-04}$*	8.6	$2.6 x10^{-05}$*	15	$1.4 x10^{-7}$*
Type: Exposure	5.6	$7.8 x10^{-04}$*	3.5	$2.3 x10^{-03}$*	4.9	$1.2 x10^{-4}$*
Month: Treatment	0.5	0.61	3.2	$5.2 x10^{-02}$	16	$1.5 x10^{-5}$*
Type: Month: Exposure	2.5	0.025*	3.4	$3.9 x10^{-03}$*	4.4	$6.2 x10^{-4}$*
