# Supplementary material for: The composition of bacterial communities associated with plastic biofilms differs between different polymers and stages of biofilm succession
Source: PLoS One. 2019 Jun 5;14(6):e0217165. doi: 10.1371/journal.pone.0217165 (PMC6550384; doi:10.1371/journal.pone.0217165)
Supplement: S2 Table — * indicates significant differences at p<0.05. (PDF) [file pone.0217165.s010.pdf]

		Df	SumsOfSqs	MeanSqs	F.Model	R2	Pr(>F)
With autotrophs	Type	6	5.12	0.85	16.1	0.24	0.001*
	Exposure	1	2.08	2.08	39.3	0.09	0.001*
	Month	2	5.08	2.54	48.1	0.24	0.001*
	Type: Exposure	5	1.28	0.26	4.83	0.06	0.001*
	Type: Month	12	3.02	0.25	4.76	0.14	0.001*
	Exposure: Month	2	1.25	0.62	11.8	0.06	0.001*
	Type: Exposure: Month	10	1.68	0.17	3.18	0.08	0.001*
	Residuals	35	1.85	0.05		0.09	
	Total	73	21.4			1	
Without autotrophs	Type	6	5.34	0.89	17.6	0.25	0.001*
	Exposure	1	1.09	1.09	21.7	0.051	0.001*
	Month	2	5.75	2.88	56.8	0.27	0.001*
	Type: Exposure	5	1.09	0.22	4.34	0.05	0.001*
	Type: Month	12	3.36	0.28	5.54	0.16	0.001*
	Exposure: Month	2	1.15	0.57	11.3	0.05	0.001*
	Type: Exposure: Month	10	1.59	0.16	3.16	0.08	0.001*
	Residuals	35	1.77	0.05		0.08	
	Total	73	21.2			1	
