# Supplementary material for: The composition of bacterial communities associated with plastic biofilms differs between different polymers and stages of biofilm succession
Source: PLoS One. 2019 Jun 5;14(6):e0217165. doi: 10.1371/journal.pone.0217165 (PMC6550384; doi:10.1371/journal.pone.0217165)
Supplement: S3 Table — The Jaccard index was used as the distance measurement. (PDF) [file pone.0217165.s011.pdf]

		Df	SumsOfSqs	MeanSqs	F.Model	R2	Pr(>F)
**After 1 week**	Type	5	2.5575	0.51149	4.5796	0.34348	0.001*
	Exposure	1	1.5434	1.54342	13.819	0.20729	0.001*
	Type: Exposure	5	2.0046	0.40092	3.5896	0.26923	0.001*
	Residuals	12	1.3403	0.11169		0.18	
	Total	23	7.4457			1	
**After 1 month**	Type	5	1.7524	0.35047	3.023	0.32959	0.001*
	Exposure	1	0.9785	0.9785	8.4402	0.18404	0.001*
	Type: Exposure	5	1.3106	0.26212	2.261	0.24651	0.001*
	Residuals	11	1.2753	0.11593		0.23986	
	Total	22	5.3167			1	
**After 2 months**	Type	5	2.0168	0.40336	3.0467	0.33781	0.001*
	Exposure	1	1.3456	1.34555	10.1635	0.22538	0.001*
	Type: Exposure	5	1.284	0.2568	1.9397	0.21507	0.003*
	Residuals	10	1.3239	0.13239		0.22175	
	Total	21	5.9703			1	

* indicates significant differences ($p<0.05$).
