# Supplementary material for: The composition of bacterial communities associated with plastic biofilms differs between different polymers and stages of biofilm succession
Source: PLoS One. 2019 Jun 5;14(6):e0217165. doi: 10.1371/journal.pone.0217165 (PMC6550384; doi:10.1371/journal.pone.0217165)
Supplement: S4 Table — Bray-Curtis dissimilarity was used as the distance measurement. (PDF) [file pone.0217165.s012.pdf]

		Df	SumsOfSqs	MeanSqs	F.Model	R2	Pr(>F)
**After 1 week**	Type	5	2.193	0.43861	8.9118	0.38689	0.001*
	Exposure	1	1.4278	1.42779	29.0102	0.25189	0.001*
	Type: Exposure	5	1.4569	0.29139	5.9205	0.25703	0.001*
	Residuals	12	0.5906	0.04922		0.10419	
	Total	23	5.6684			1	
**After 1 month**	Type	5	1.2345	0.24689	4.613	0.37296	0.001*
	Exposure	1	0.712	0.71205	13.3043	0.21513	0.001*
	Type: Exposure	5	0.7747	0.15493	2.8949	0.23405	0.001*
	Residuals	11	0.5887	0.05352		0.17787	
	Total	22	3.3099			1	
**After 2 months**	Type	5	1.6556	0.33113	5.1858	0.39508	0.001*
	Exposure	1	1.1561	1.15607	18.1051	0.27587	0.001*
	Type: Exposure	5	0.7404	0.14809	2.3192	0.17669	0.007*
	Residuals	10	0.6385	0.06385		0.15237	
	Total	21	4.1907			1	

* indicates significant differences ($p<0.05$).
