# Supplementary material for: The composition of bacterial communities associated with plastic biofilms differs between different polymers and stages of biofilm succession
Source: PLoS One. 2019 Jun 5;14(6):e0217165. doi: 10.1371/journal.pone.0217165 (PMC6550384; doi:10.1371/journal.pone.0217165)
Supplement: S4 Fig — OTUs representing < 5% of the total bacterial community were grouped into the category “Others”. The taxonomic classification of OTUs was obtained using a 97% similarity of the partial 16S rRNA gene and the SILVA database. (PDF) [file pone.0217165.s020.pdf]

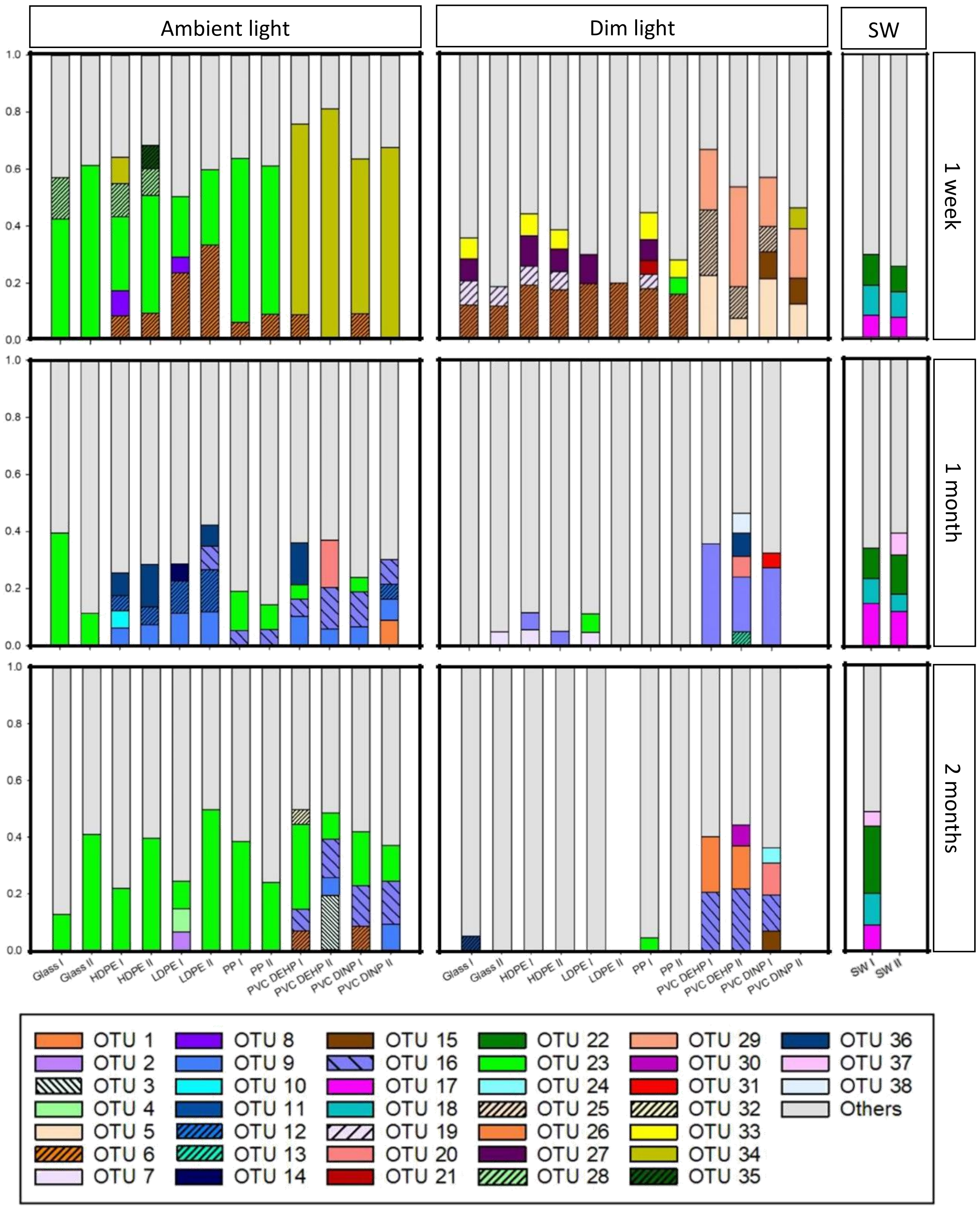

Ambient light
Dim light
SW
1 week
1 month
2 months
1.0
0.8
0.6
0.4
0.2
0.0
Glass I
Glass II
HDPE I
HDPE II
LDPE I
LDPE II
PP I
PP II
PVC DEHP I
PVC DEHP II
PVC DINP I
PVC DINP II
SW I
SW II
OTU 1
OTU 2
OTU 3
OTU 4
OTU 5
OTU 6
OTU 7
OTU 8
OTU 9
OTU 10
OTU 11
OTU 12
OTU 13
OTU 14
OTU 15
OTU 16
OTU 17
OTU 18
OTU 19
OTU 20
OTU 21
OTU 22
OTU 23
OTU 24
OTU 25
OTU 26
OTU 27
OTU 28
OTU 29
OTU 30
OTU 31
OTU 32
OTU 33
OTU 34
OTU 35
OTU 36
OTU 37
OTU 38
Others

OTU1	k__Bacteria; p__Proteobacteria; c__Gammaproteobacteria; o__Pseudomonadales; f__Moraxellaceae; g__Acinetobacter; s__Acinetobacter radioresistens DSM 6976 = NBRC 102413 = CIP 103788
OTU 2	k__Bacteria; p__Bacteroidetes; c__Flavobacteriia; o__Flavobacteriales; f__Flavobacteriaceae; g__Flaviramulus; s__marine bacterium J20-17
OTU 3<br>OTU 4	k__Bacteria; p__Proteobacteria; c__Alphaproteobacteria; o__Caulobacterales; f__Hyphomonadaceae; g__Robiginitomaculum; s__uncultured alpha proteobacterium<br>k__Bacteria; p__Cyanobacteria; c__Cyanobacteria; o__SubsectionIII; f__FamilyI; g__Phormidium; s__uncultured bacterium
OTU 5	k__Bacteria; p__Proteobacteria; c__Gammaproteobacteria; o__Oceanospirillales; f__Oceanospirillaceae; g__uncultured; s__uncultured bacterium
OTU 6	k__Bacteria; p__Proteobacteria; c__Gammaproteobacteria; o__Oceanospirillales; f__Oleiphilaceae; g__Oleiphilus; s__uncultured bacterium AD12-E7
OTU 7	k__Bacteria; p__Bacteroidetes; c__Flavobacteriia; o__Flavobacteriales; f__Flavobacteriaceae; g__Aquibacter; s__uncultured bacterium
OTU 8	k__Bacteria; p__Bacteroidetes; c__Flavobacteriia; o__Flavobacteriales; f__Flavobacteriaceae; g__Mesoflavibacter; s__Mesoflavibacter sp. NBRC 110217
OTU 9	k__Bacteria; p__Proteobacteria; c__Alphaproteobacteria; o__Rhodobacterales; f__Rhodobacteraceae; g__Loktanella; s__uncultured alpha proteobacterium
OTU 10	k__Bacteria; p__Proteobacteria; c__Alphaproteobacteria; o__Sphingomonadales; f__Erythrobacteraceae; g__Erythrobacter; s__uncultured bacterium
OTU 11	k__Bacteria; p__Proteobacteria; c__Alphaproteobacteria; o__Rhizobiales; f__Phyllobacteriaceae; g__Pseudahrensia; s__uncultured marine bacterium
OTU 12	k__Bacteria; p__Proteobacteria; c__Alphaproteobacteria; o__Rhodobacterales; f__Rhodobacteraceae; g__Ruegeria; s__uncultured Ruegeria sp.
OTU 13	k__Bacteria; p__Proteobacteria; c__Alphaproteobacteria; o__Alphaproteobacteria Incertae Sedis; f__Unknown Family; g__uncultured; s__uncultured bacterium
OTU 14	k__Bacteria; p__Proteobacteria; c__Alphaproteobacteria; o__Rhizobiales; f__Phyllobacteriaceae; g__Hoeflea; s__uncultured bacterium
OTU 15	k__Bacteria; p__Proteobacteria; c__Gammaproteobacteria; o__Cellvibrionales; f__Cellvibrionaceae; g__Aestuariicella; s__Proteobacteria
OTU 16	k__Bacteria; p__Proteobacteria; c__Alphaproteobacteria; o__Caulobacterales; f__Hyphomonadaceae; g__uncultured; s__uncultured bacterium
OTU 17<br>OTU 18	k__Bacteria; p__Actinobacteria; c__Acidimicrobiia; o__Acidimicrobiales; f__OM1 clade; g__Candidatus Actinomarina; s__uncultured bacterium<br>k__Bacteria; p__Proteobacteria; c__Alphaproteobacteria; o__SAR11 clade; f__Surface 1; g__uncultured bacterium
OTU 19	k__Bacteria; p__Bacteroidetes; c__Flavobacteriia; o__Flavobacteriales; f__Flavobacteriaceae; g__Pseudofulvibacter;

	s__Pseudofulvibacter geojedonensis
OTU 20	k__Bacteria; p__Proteobacteria; c__Gammaproteobacteria; o__Arenicellales; f__Arenicellaceae; g__Arenicella; s__uncultured proteobacterium
OTU 21	k__Bacteria; p__Proteobacteria; c__Gammaproteobacteria; o__Oceanospirillales; f__Oleiphilaceae; g__Oleiphilus; s__Oleiphilus sp. HI0081
OTU 22	k__Bacteria; p__Cyanobacteria; c__Cyanobacteria; o__SubsectionI; f__FamilyI; g__Synechococcus; s__uncultured bacterium
OTU 23	k__Bacteria; p__Cyanobacteria; c__Chloroplast; o__uncultured cyanobacterium
OTU 24	k__Bacteria; p__Proteobacteria; c__Alphaproteobacteria; o__Rhizobiales; f__Rhodobiaceae; g__uncultured; s__uncultured bacterium
OTU 25	k__Bacteria; p__Proteobacteria; c__Gammaproteobacteria; o__Cellvibrionales; f__Cellvibrionaceae; g__Simiduia; s__uncultured bacterium
OTU 26	k__Bacteria; p__Proteobacteria; c__Gammaproteobacteria; o__Cellvibrionales; f__Cellvibrionaceae; g__uncultured; s__uncultured bacterium
OTU 27	k__Bacteria; p__Bacteroidetes; c__Flavobacteriia; o__Flavobacteriales; f__Flavobacteriaceae; g__Tenacibaculum; s__Tenacibaculum aiptasiae
OTU 28	k__Bacteria; p__Cyanobacteria; c__Chloroplast; o__uncultured bacterium
OTU 29	k__Bacteria; p__Proteobacteria; c__Gammaproteobacteria; o__Oceanospirillales; f__Oceanospirillaceae; g__Spongiispira; s__Spongiispira norvegica
OTU 30	k__Bacteria; p__Bacteroidetes; c__Flavobacteriia; o__Flavobacteriales; f__Flavobacteriaceae; g__Leptobacterium; s__uncultured bacterium
OTU 31	k__Bacteria; p__Proteobacteria; c__Deltaproteobacteria; o__Myxococcales; f__Nannocystaceae; g__uncultured; s__uncultured proteobacterium
OTU 32	k__Bacteria; p__Proteobacteria; c__Gammaproteobacteria; o__Oceanospirillales; f__Oleiphilaceae; g__Oleiphilus; s__uncultured Oleiphilus sp.
OTU 33	k__Bacteria; p__Proteobacteria; c__Gammaproteobacteria; o__Oceanospirillales; f__SS1-B-06-26; g__uncultured bacterium
OTU 34	k__Bacteria; p__Proteobacteria; c__Gammaproteobacteria; o__Alteromonadales; f__Alteromonadaceae; g__Alteromonas; s__uncultured bacterium
OTU 35	k__Bacteria; p__Cyanobacteria; c__Chloroplast; o__Virgulinella fragilis
OTU 36	k__Bacteria; p__Proteobacteria; c__Alphaproteobacteria; o__Rhodobacterales; f__Rhodobacteraceae; g__Pseudophaeobacter; s__Phaeobacter sp. 11ANDIMAR09
OTU 37	k__Bacteria; p__Bacteroidetes; c__Flavobacteriia; o__Flavobacteriales; f__Flavobacteriaceae; g__Polaribacter 4; s__uncultured bacterium
OTU 38	k__Bacteria; p__Proteobacteria; c__Alphaproteobacteria; o__Sphin
